# Selection of GmSWEET39 for oil and protein improvement in soybean

**DOI:** 10.1371/journal.pgen.1009114

**Published:** 2020-11-11

**Authors:** Hengyou Zhang, Wolfgang Goettel, Qijian Song, He Jiang, Zhenbin Hu, Ming Li Wang, Yong-qiang Charles An

**Affiliations:** 1 Donald Danforth Plant Science Center, St. Louis, MO, United States of America; 2 US Department of Agriculture, Agricultural Research Service, Soybean Genomics and Improvement Laboratory, Beltsville, MD, United States of America; 3 US Department of Agriculture, Agricultural Research Service, Plant Genetics Resource Conservation Unit, Griffin, GA, United States of America; 4 US Department of Agriculture, Agricultural Research Service, Plant Genetics Research Unit at Donald Danforth Plant Science Center, St. Louis, MO, United States of America; Nanjing Agricultural University, CHINA

## Abstract

Soybean [*Glycine max* (L.) Merr.] was domesticated from wild soybean (*G*. *soja* Sieb. and Zucc.) and has been further improved as a dual-use seed crop to provide highly valuable oil and protein for food, feed, and industrial applications. However, the underlying genetic and molecular basis remains less understood. Having combined high-confidence bi-parental linkage mapping with high-resolution association analysis based on 631 whole sequenced genomes, we mapped major soybean protein and oil QTLs on chromosome15 to a sugar transporter gene (*GmSWEET39*). A two-nucleotide CC deletion truncating C-terminus of *GmSWEET39* was strongly associated with high seed oil and low seed protein, suggesting its pleiotropic effect on protein and oil content. *GmSWEET39* was predominantly expressed in parenchyma and integument of the seed coat, and likely regulates oil and protein accumulation by affecting sugar delivery from maternal seed coat to the filial embryo. We demonstrated that *GmSWEET39* has a dual function for both oil and protein improvement and undergoes two different paths of artificial selection. A CC deletion (CC-) haplotype H1 has been intensively selected during domestication and extensively used in soybean improvement worldwide. H1 is fixed in North American soybean cultivars. The protein-favored (CC+) haplotype H3 still undergoes ongoing selection, reflecting its sustainable role for soybean protein improvement. The comprehensive knowledge on the molecular basis underlying the major QTL and *GmSWEET39* haplotypes associated with soybean improvement would be valuable to design new strategies for soybean seed quality improvement using molecular breeding and biotechnological approaches.

## Author summary

We map highly effective protein and oil QTLs to a seed coat-preferentially expressed sugar transporter (GmSWEET39) gene by a combination of association analysis based on 631 whole-genome sequencing data and a bi-parental linkage mapping, proving that *GmSWEET39* has pleiotropic associations with two of the most important soybean traits, seed protein and oil. A 2-bp (CC) deletion in *GmSWEET39* is associated with higher seed oil and lower seed protein, and has been extensively selected and used worldwide, likely for higher oil. The intensive use or fixation of the CC deletion in soybean breeding result in low protein in current soybean cultivars, which is a big problem facing the current soybean industry. The knowledge about the genetic basis and identification of two major haplotypes for high protein and high oil should be highly significant to address the issue related to low protein in the current soybean industry and meet dramatically increasing need for plant-based protein in the food industry. Our successful integrative and "big-data-driven” approach, which uses the huge amount of genome and transcriptome sequencing and phenotypic data available in the community, should provide an effective case study for post-genomic data-driven research.

## Introduction

Cultivated soybean [*Glycine max* (L.) Merr.] is an important seed crop grown worldwide. It was domesticated from wild soybean (*G*. *soja* Sieb. and Zucc.) in East Asia approximately 6,000–9,000 years ago and has been further improved through breeding as a major oilseed crop to provide a source of both valuable vegetable oil and protein for uses in food, feed, and industrial applications [1]. Unlike in Asia where farmers grew locally adapted landraces and selections of them for thousands of years, soybean has a short history in North America, and it was introduced to North America in 1765. Soybean had been mostly grown as a forage crop in North America for the next 155 years until the importance of soybeans as a valuable source of protein and oil was realized at the beginning of the 20th century. Starting from the 1920s, soybean has been grown as a seed/grain crop mainly for seed protein and oil. Soybean yield (bushels per acre) nearly doubled since 1987 [2], making soybean one of the sustainable crops to meet the rapid demand for plant-based protein and oil for a continuously increasing world population [3]. However, it was often observed that protein content in seeds is quantitatively inherited and negatively correlated with seed oil content and yield [4–6], making it challenging to increase soybean protein content while maintaining the desired seed oil level and seed yield.

In the past decades, over 300 quantitative trait loci (QTLs) controlling soybean seed oil and protein content have been identified using accessions with the different genetic background [7], suggesting that soybean oil and protein content are subject to a highly diverse and complex genetic control. Although great efforts have been dedicated to utilizing diverse genetic resources to develop cultivars containing both improved protein and oil traits or improve one trait without a negative impact on the other trait, progress has been limited. In contrast, it was observed that US commercial cultivars released over the past several decades contain decreased seed protein content (http://unitedsoybean.org/), which is likely an unintended consequence of a strong selection for high oil. To effectively exploit the soybean diversity to improve the seed traits with no or little negative impact on the others, it is crucial to identify the causative genes/alleles for major QTLs and illustrate the molecular basis underlying their associated trait interaction and genetic diversity in soybean domestication and breeding.

In the present study, having applied a combination of high confidence bi-parental linkage mapping using a population of recombinant inbred lines (RIL) and high-resolution association analysis using 631 diverse whole genome sequences, we mapped a major protein and oil content QTL on chr15 to a sugar transporter gene and demonstrated that the sugar transporter gene was preferentially expressed in seed coat tissues. We further revealed that a 2-bp (CC)-deletion in the gene was associated with the variation of seed oil and protein content. The CC deletion was selected during soybean domestication and improvement. Haplotype analyses showed that a CC deletion-carrying haplotype is widely present in cultivated soybeans grown worldwide and has been fixed in US modern soybean breeding, highlighting the significance of the high-oil CC-deletion haplotype in soybean improvement. The CC-deletion allele also contributes to low protein in modern soybean cultivars, a big problem facing the current soybean industry. Our result will greatly advance our understanding of these complex seed quality traits and benefit seed improvement in soybean.

## Results

### Linkage analyses identified a major QTL region controlling both seed oil and protein content

**Fig 1:**
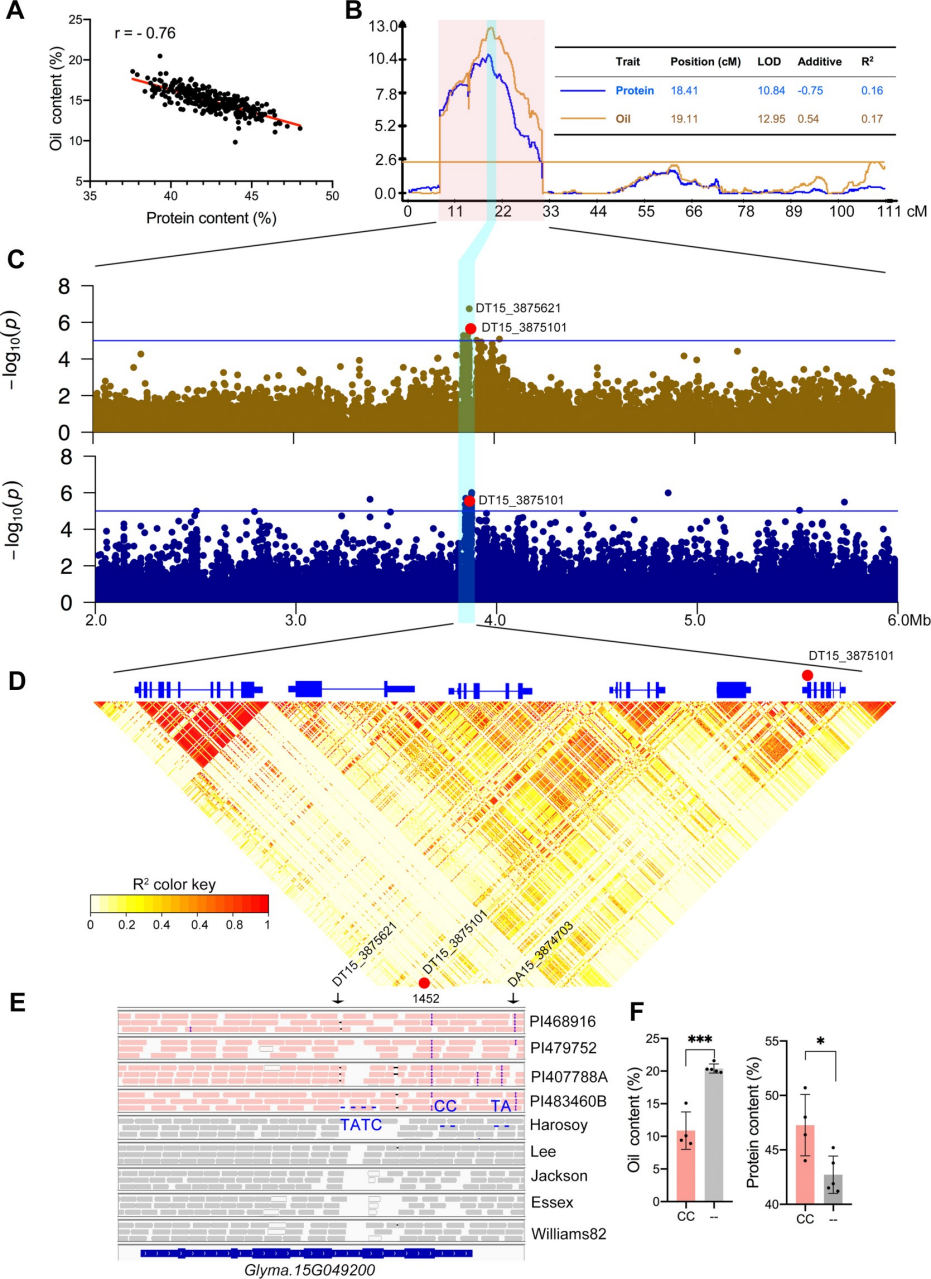
Identification of the major QTL on chromosome 15 and its underlying candidate gene. (**A**) Seed oil and protein from RILs exhibited a negative correlation. (**B**) Linkage mapping using RILs identified two overlapping QTLs on Chr15 for oil and protein, respectively. (**C**) Regional association analysis identified a cluster of variants significantly associated with oil and protein content. The red solid dot represents a significant association (DT15_3875101). (**D**) Six gene models and the LD pattern within the significant 34-kb region. (**E**) IGV view of variant patterns between high protein and low protein soybean genotypes. The two variants occurring in the non-coding region were indicated with arrows in black. Variant patterns at the three sites were shown as TATC/--- (alternative/reference), --/CC, and--/TA. (**F**) The CC deletion (- -) at DT15_3875101 is highly correlated with high-oil, and low-protein content. CC presence is indicated by CC. * and *** indicate the significance at *p*< 0.05 and *p* < 0.001, respectively.

A population of 300 RILs was used for linkage analyses. The two parental lines exhibited significant differences in seed oil and protein content, with Williams82 (*G*. *max*) seeds containing 39.33% protein and 20.47% oil content, and PI479752 (*G*. *soja*) containing 43.99% protein and 9.82% oil content (**S1A Fig**). RILs varied widely in seed oil (9.82–20.47%) and protein (37.64–47.99%), and protein and oil content were negatively correlated in the RILs (*r* = -0.76) (**Fig 1A**). Broad-sense heritability (H^2^) of oil and protein in the RILs across two-year tests were 0.87 and 0.82, respectively, indicating that both traits were mainly controlled genetically which allows a substantial genetic improvement of oil and protein content.

Composite interval mapping with the SoySNP50K genotyping data identified two major QTLs on chr15 and chr20 (**S1C Fig**). We focused on the QTL on chr15 in this study. Two overlapping QTLs, *qSeedOil_15* and *qSeedPro_15*, for oil (LOD = 12.95) and protein (LOD = 10.84) were identified respectively (**Fig 1B**). *qSeedOil_15* was flanked by two markers Gm15_2391075_G_A and Gm15_6028005_A_G (8.94–29.10 cM), while *qSeedPro_15* was flanked by Gm15_1989918_T_G and Gm15_5829758_G_T (7.18–28.21 cM). Both QTLs physically spanned approximately 4 Mb (physical interval, chr15: 2.0–6.0 Mb) and 92.0% of the two QTL intervals overlapped. This region coincided with previously-published major QTLs for seed oil or protein content in soybean [4, 8–11], indicating that this region contained a major QTL gene(s) contributing significantly to the large variation in both protein and oil content.

### A sugar transporter gene highly associated with the seed protein and oil QTL

To uncover the gene(s) underlying *qSeedOil_15* and *qSeedPro_15*, we carried out association analyses of the two traits with DNA variants within the 4 Mb QTL region using a panel of 631 diverse wild and cultivated soybean accessions collected worldwide. Accessions in this panel had a wide variation in seed protein (32.5–52.3%) and oil content (10.1–23.5%). Overall, the two traits were significantly negatively correlated (*r* = -0.62) (**S1B Fig**). We analyzed the whole genome sequences of the 631 accessions and identified a total of 96,813 DNA variants including 79,725 single nucleotide polymorphisms (SNPs) and 17,088 insertions and deletions (indels) in this 4-Mb region. These DNA variants in the region were tested for their associations with oil and protein. The association analysis revealed that three most significant indels (DT15_3875621, DT15_3875101, DA15_3874703) for oil content located in a cluster of DNA variants spanning a 34-kb region that was highly significant (*p* < 1e-5) for seed oil in this panel (**Fig 1C**). The three most significant indels were also highly significant for protein content (**S2 Table**). This finding was consistent with the observation that the two QTLs were highly overlapped in the linkage mapping analysis described above (**Fig 1B**), strongly suggesting that seed oil and protein might be controlled by the same locus.

The three most significant variants were all located within the genomic region of *Glyma*.*15G049200*. The indel DT15_3875101 (--/CC, reference/alternative allele) located in the coding region (exon 6), while the other two, DT15_3875621 (TATC/----) and DA15_3874703 (--/TA), located in the 4th intron and the 3’ untranslated region (UTR) respectively. The CC indel was polymorphic in two parents of the RIL population. The high-oil and low-protein parent (Williams82) contained the CC deletion (CC-) that was not present in the low-oil and high-protein parent (PI479752). The CC deletion in the coding sequence (CDS) of *Glyma*.*15G049200* caused a frameshift (see below). We examined *Glyma*.*15G049200* genomic sequences of 631 accessions in the association panel and identified two *G*. *max* accessions containing the gene sequence identical to Williams82-type allele except for the CC deletion. The two accessions contained significantly lower oil content and higher protein in seeds than accessions carrying the Williams82-type allele (*p* < 0.05, see haplotype analysis section, H1 vs H2, **Fig 4A and 4B**), confirming that the CC indel rather than the other two non-CDS DNA variants was causative of oil and protein variation. We also examined the parental lines (PI468916, PI407788A, PI483460B and Williams82) of bi-parental linkage mapping populations whose protein and oil QTLs were mapped to the genomic regions overlapped with *qSeedOil_15* and *qSeedPro_15* [8, 10, 12]. Sequence alignments revealed that DT15_3875101 in exon 6 was the only variant in *Glyma*.*15G049200* among these accessions fully associated with oil and protein content (**Fig 1E**). Notably, seeds of these accessions with the CC presence (CC+) in *Glyma*.*15G049200* contained 9.5% less oil (*p*<0.001) and 4.6% more protein (*p*<0.05) than those with the CC deletion (CC-) (**Fig 1F**). Thus, the CC InDel, not the other two DNA variants, is the causative allele for variation of oil and protein content. In addition, *Glyma*.*15G049200* was the only one of the six genes within the linkage disequilibrium block showing predominant expression in developing seeds (**Fig 1D**, **S2A** and **S2B Fig**), implying that the role of *Glyma*.*15G049200* was specific to seed filling. *Glyma*.*15G049200* encoded an ortholog of the *Arabidopsis* AtSWEET15 sugar transporter (AT5G13170) that also plays a role in lipid accumulation in seeds [13]. Thus, all evidence suggests that *Glyma*.*15G049200* underlies the QTL on chr15 controlling both protein and oil content and InDel DT15_3875101 in *Glyma*.*15G049200* is the causative allele for protein and oil content variation.

### The CC deletion causes the truncated *GmSWEET39*

**Fig 2:**
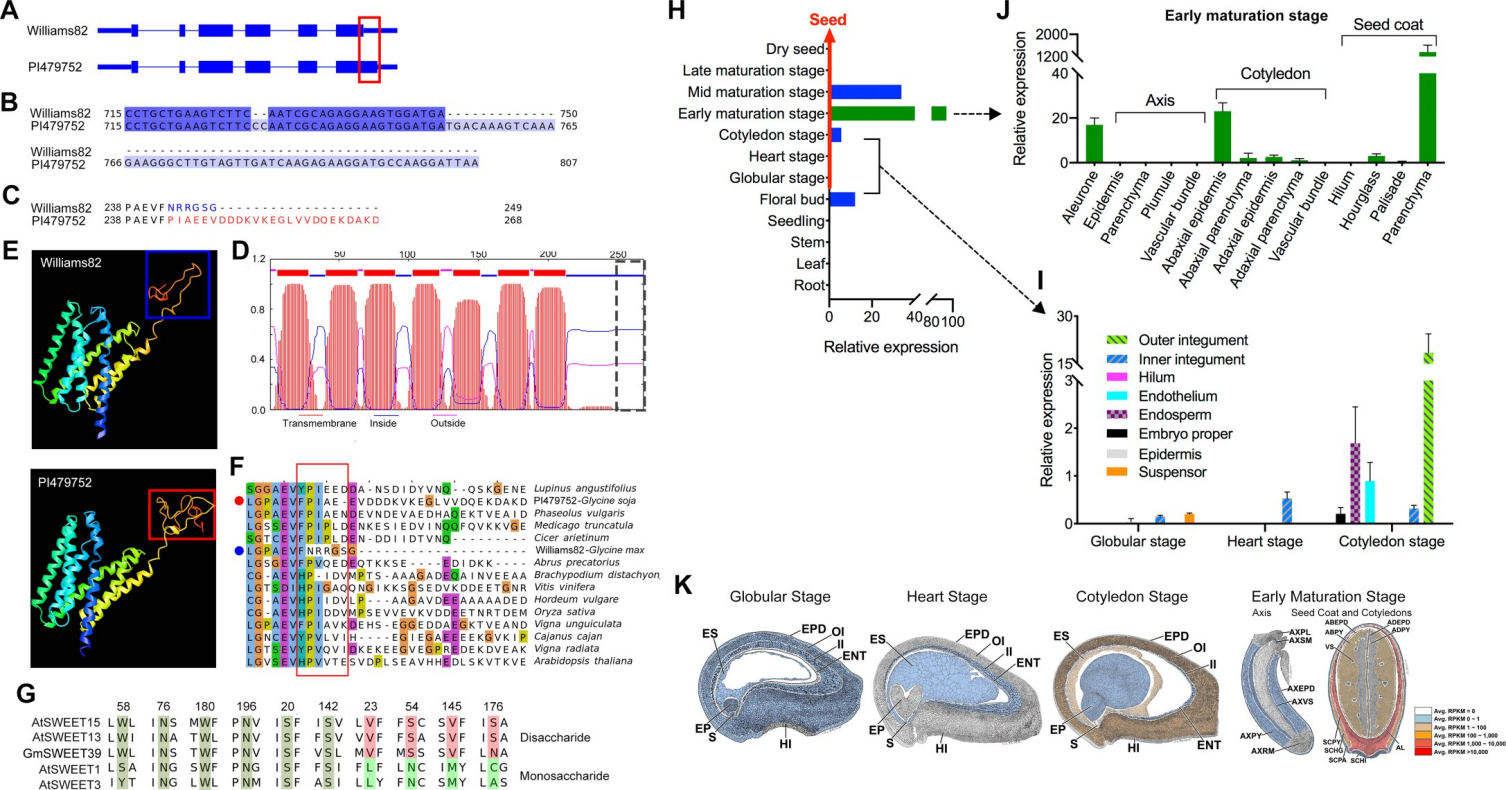
Sequence and expression analysis of *GmSWEET39* alleles. (**A**) A schematic diagram shows the sequence variation that occurs in the 6th exon between the two parental lines of the RIL population, Willams82 and PI479752. (**B**) The CC deletion in Williams82 causes a reading frameshift and a truncated protein at the C-terminus. (**C**) GmSWEET39 is 19 amino acid shorter in Williams82 than it in PI479752. (**D**) Predicted transmembrane domains in GmSWEET39. The sequence differences as shown in (**B**) and (**C**) were highlighted with the gray dotted rectangle. (**E**) Comparison of the 3D protein structure between Willams82 and PI479752 GmSWEET39 proteins. The difference in the structure at the C-terminal tails is highlighted with rectangles in blue and red. (**F**) Sequence alignment between the C-terminal peptides of the SWEET homologs from different species including legumes. The putatively conserved region was highlighted with the rectangle in red. (**G**) GmSWEET39 is conserved at the amino acids essential for disaccharide transport. (**H**) A bar plot showing the expression pattern of *GmSWEET39* in different soybean tissues and the whole seeds at different maturation stages. (**I**) Relative expression of *GmSWEET39* in different subregions of developing seeds of soybean cultivar Williams 82 (CC deletion) at globular, heart, and cotyledon stages. (**J**) Relative expression pattern of CC-deleted *GmSWEET39* in the sub-regions in developing seeds of Williams 82 at the early maturation stage. (**K**) Cartoon depicting the expression patterns of *GmSWEET39* in different tissues/compartments of globular, heart, cotyledon, and early maturation seeds of Williams 82 (CC deletion). Abbreviation: AB—Abaxial; AD—Adaxial; AL—Aleurone; AX—Axis; Cot—Cotyledon; EP—Embryo Proper; EPD—Epidermis; ENT—Endothelium; ES—Endosperm; FBUD—Floral Bud; HG—Hourglass; HI—Hilum; II—Inner Integument; OI—Outer Integument; PA—Palisade; PL—Plumule; PY—Parenchyma; RM—Root Meristem; S—Suspensor; SC—Seed Coat; SM—Shoot Meristem; VS—Vascular Bundle. The seed images were acquired from http://seedgenenetwork.net/sequence.

*Glyma*.*15G049200* was previously assigned as *GmSWEET39* in a *SWEET* (Sugars Will Eventually be Exported Transporters) gene family analysis [14]. The CC indel (DT15_3875101) occurred in the coding region and caused a reading frameshift starting from the 730th nucleotide of GmSWEET39. The frameshift resulted in a premature stop codon and six amino acid changes at the C-terminus of GmSWEET39 in Williams82 (**Fig 2A** and **2B**). Thus, the CC deletion truncated 19 amino acids from the C-terminus of GmSWEET39 in Williams82 compared with the intact GmSWEET39 in PI479752 (**Fig 2C**). Like its orthologous *AtSWEET15* gene [15, 16], intact *GmSWEET39* encoded a sugar transporter protein with seven predicted transmembrane (TM) domains and a cytoplasmic C-terminal tail (**Fig 2D**). Lack of the 19 C-terminal amino acids and the C-terminal amino acid changes in Williams82-type GmSWEET39 caused no obvious change in the predicted 3-D protein structure of the seventh TM domain compared to GmSWEET39 in PI479752 (**Fig 2E**). However, the reading frameshift changed three amino acids in the cytoplasmic C-terminal tail that appeared to be highly conserved in legumes (**Fig 2F**). Sequence alignment showed conservation between GmSWEET39 and two AtSWEETs (AtSWEET13, AtSWEET15) in four key amino acids that were specific to disaccharide transport [15], suggesting the potential role of GmSWEET39 in transporting disaccharides (**Fig 2G**). It remains to be determined how the amino acid sequence change and loss of the 19 amino acids affect the activity of GmSWEET39 and impact oil and protein accumulation in seeds.

### *GmSWEET39* encodes a sugar transporter preferentially expressed in developing seed coat

We examined expression patterns of *GmSWEET39* (CC-) in soybean major organs and different seed compartments of Williams 82 at different seed development stages using the Goldberg-Harada Soybean RNA-Seq Dataset (http://seedgenenetwork.net/soybean). Interestingly, *GmSWEET39* was predominantly expressed in the floral bud and developing seeds (**Fig 2H**). Briefly, the accumulation of *GmSWEET39* transcripts in seeds was detected as early as the globular stage and reached the highest level at the early maturation stage over the course of seed development. The transcript abundance significantly decreased after seeds reached the early maturation stage and it was undetectable in the late maturation and dry seed stages. Before the early maturation stage, transcripts of *GmSWEET39* began to accumulate in some subregions of the seed coat such as the inner/outer integuments during the heart and cotyledon stages (**Fig 2J**). At the early maturation stage (**Fig 2I**), transcripts of *GmSWEET39* showed a significant accumulation in subregions of the cotyledon (abaxial epidermis) and seed coat (parenchyma). Notably, the transcript abundance in the parenchyma of the seed coat at the early maturation stage was approximately 60–400 times higher than in the abaxial epidermis and outer integument at the cotyledon stage. Expression patterns of *GmSWEET39* in the aforementioned compartments of developing seeds were illustrated in **Fig 2K**. These results suggest the special role of *GmSWEET39* during seed development, especially in the parenchyma of the seed coat. In plants, parenchyma is the innermost part of the seed coat with direct contact with the endosperm [17]. This layer is crucial in assimilate unloading from the maternal seed coat and in the initiation of the storage phase in legumes [18]. Given that *GmSWEET39* encoded a sugar transporter that is specifically expressed in seed coat subregions during seed development, it is reasonable to presume that GmSWEET39 plays an important role in facilitating sugar transfer in the seed coat to allocate photosynthetic sucrose into the embryo and that the increased sucrose accumulation enhances the subsequent sucrose-derived fatty acid biosynthesis [19, 20].

### *GmSWEET39* has been selected over soybean domestication and improvement for oil and protein improvement

**Fig 3:**
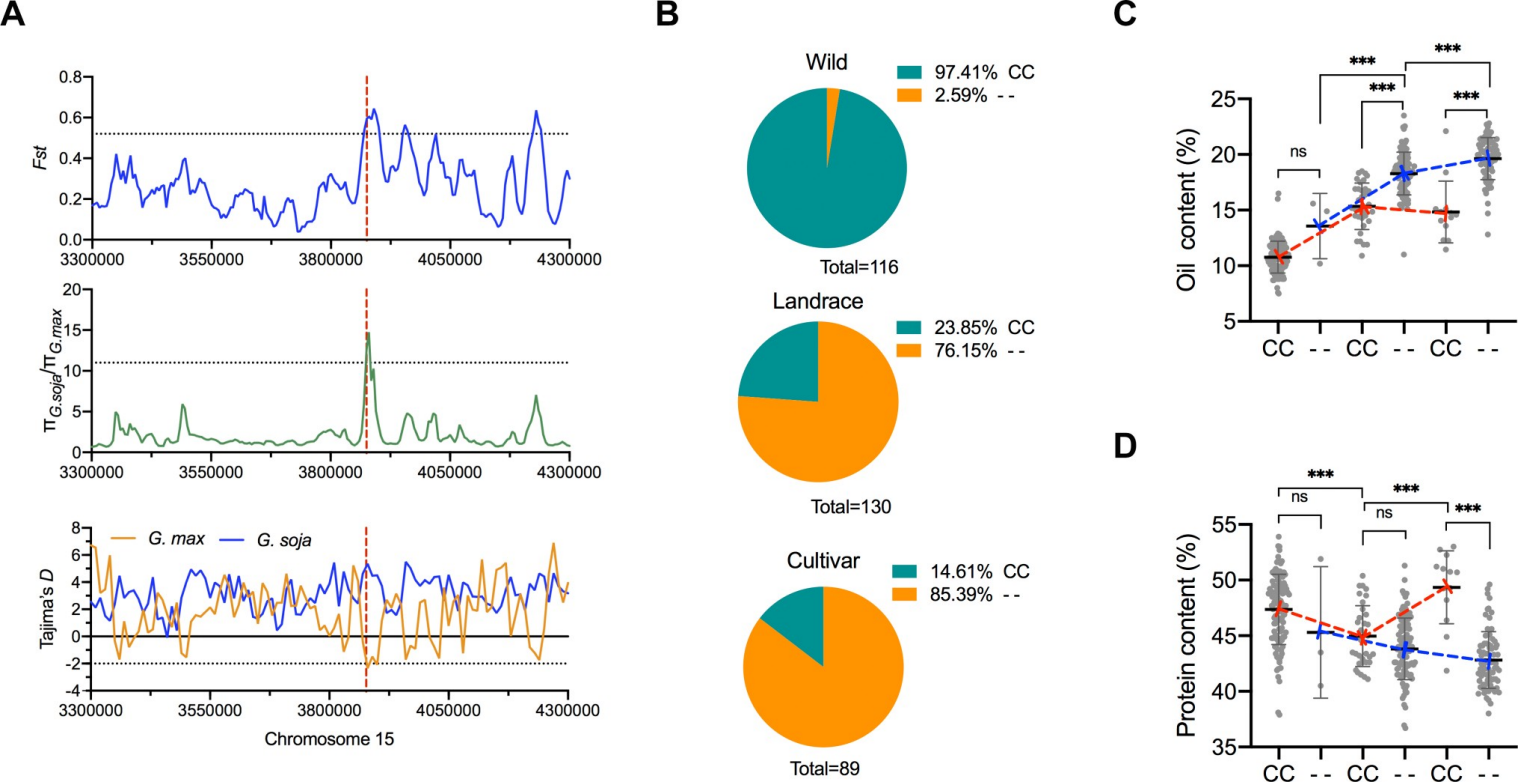
Genetic diversity and allele analysis of *GmSWEET39*. (**A**) Genetic diversity (π), Tajima’s *D*, and fixation index (*Fst*) among the three groups across the 1-Mb genomic region harboring *GmSWEET39*. The horizontal dotted line indicates the genome-wide threshold as described by Wang et al [50]. A red dashed line indicates the physical position of *GmSWEET39*. (**B**) Genotype frequency distribution of CC presence (CC) and absence (- -) in *GmSWEET39* in wild, landrace, and improved cultivar. Allelic analysis of CC presence (CC) and absence (- -) in *GmSWEET39* on oil **(C)** and protein content (**D**). *** indicates the statistical significance at *p* < 0.001. ns: not significant.

We examined the indexes of selection in the 1-Mb genomic region harboring *GmSWEET39* using the fixation index (*Fst*), diversity (π) and Tajima’s *D*. The results from these indexes all indicated that *GmSWEET39* located in an approximately 40-kb genomic region that underwent a selective sweep (**Fig 3A**), suggesting that *GmSWEET39* underwent selection during soybean domestication.

We split the panel of accessions into wild soybean (*G*. *soja*), landrace, and improved cultivar based on available germplasm type assignment and examined the allelic frequency of the CC deletion. We detected three CC- *G*. *soja* accessions. The allelic frequency of the CC deletion increased over the course of soybean domestication and improvement with 2.59% (3 of 114) in wild soybean, 76.15% (99 of 130) in landrace and 85.39% (76 of 89) in improved cultivar (**Fig 3B**). We also observed increases in oil content in CC- accessions over the courses of soybean domestication (*G*. *soja* to landrace) and improvement (landrace to cultivar) (**Fig 3C**). In CC- accessions, oil content significantly increased by 4.72% (*p* < 0.001) from *G*. *soja* to landrace and 1.35% (*p* < 0.001) from landrace to cultivars. However, oil in CC+ accessions was decreased by 0.5% during the transition from landrace to cultivars. The increasing trend of oil content in CC- accessions was consistent with that of CC- allele frequency over the transitions in *G*. *soja*-landrace-cultivar (**Fig 3C**), suggesting that the CC- allele rather than CC+ allele was selected and used for high oil during both domestication and improvement processes.

In consistence with observed negative relationship of oil and protein content, protein content reduced in CC- accessions during the domestication and improvement (**Fig 3D**). Protein content was decreased in CC+ accessions from *G*. *soja* to landrace. On the contrary, protein content was significantly increased by 4.38% (*p* < 0.001) in CC+ accessions during the transition from landrace to cultivar. This finding with protein changes suggested that CC+ allele of *GmSWEET39* might be selected and used for developing high protein cultivars during the improvement process. These observations implied that soybean might experience two paths for soybean oil and protein improvement using the two distinct alleles of *GmSWEET39*: CC- has been selected/used for oil improvement the over the courses of soybean domestication and improvement while CC+ allele has been selected/used for developing high protein cultivars during the improvement of *G*. *max* accessions.

We further examined oil and protein content difference between CC- and CC+ accessions within each germplasm group. Oil was averagely higher in CC- than CC+ accessions within wild (2.8% increase), landrace (2.94% increase, *p* < 0.001), and cultivar (4.80% increase, *p* < 0.001) (**Fig 3C**). Accordingly, protein content in CC- accessions was lower than CC+ accessions within each of the three groups (2.70% decrease in wild, 1.15% decrease in landrace) with the greatest difference seen in cultivars (6.53% decrease, *p* < 0.001) (**Fig 3D**). These results were supportive of the pleiotropic roles of *GmSWEET39* on oil and protein. Notably, more dramatic differences for both oil and protein content between CC- and CC+ accessions in cultivars than landrace were observed. This could be the result of differentially integrating oil-favor alleles and protein-favor alleles of other QTLs in CC- and CC+ accessions during soybean improvement for high oil and high protein cultivars, which augment the difference of CC- and CC- cultivars in protein and oil.

### Discriminatory uses of the two major haplotypes in oil and protein improvement

**Fig 4:**
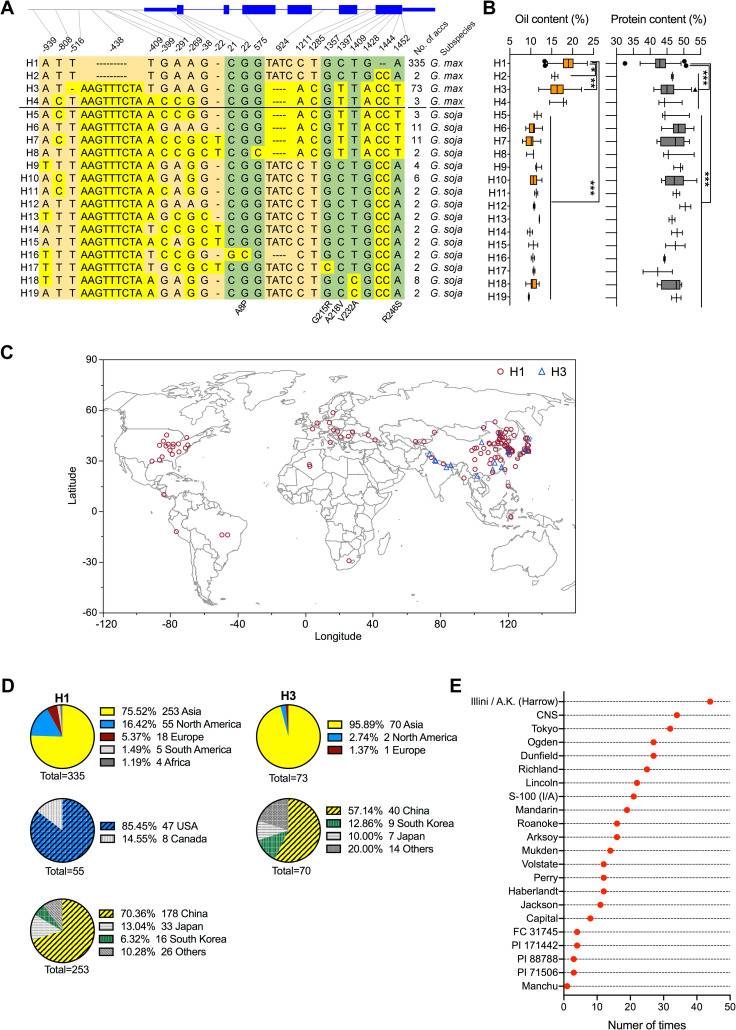
Analyses of haplotype distribution worldwide and the use frequency of H1 in US soybean breeding. (**A**) A schematic diagram shows the distribution of all DNA variants identified in *GmSWEET39* and its putative 1,000-bp promoter region. 19 haplotypes (H) were illustrated in the lower panel. The number of accessions carrying the corresponding haplotypes and the subspecies categorization was indicated beside the panel **(B)** Phenotypic comparison (oil and protein) among the 19 haplotypes. *, **, and *** indicate the statistical significance at *p* < 0.05, *p* < 0.01, and *p* < 0.001, respectively. (**C**) Geographic distribution of two major haplotypes (H1, H3) across the world. (**D**) Pie charts show the percentage of the origin of the two haplotypes, H1 and H3, important for oil content and protein content, respectively. The top pie chart in each haplotype shows the overall distribution by continent. The lower charts indicate detailed information for Asia and North America. The percentage, amount of accessions, and origin per haplotype are indicated next to the color representation in the legend. (**E**) Number of times that the 22 representative cultivars carrying the CC deletion used in breeding soybean cultivars. Y axis indicates the number of times that the corresponding cultivar has been used for soybean breeding.

We performed a haplotype analysis for the accessions used in the association panel by examining the sequence diversity of the transcribed genic region of *GmSWEET39* and the 1.0 kb region upstream of the start codon (ATG). Based on patterns of the fragment variants, we identified 19 haplotypes with each detected in at least two accessions (**Fig 4A, S3 Table**). H1-H4 haplotypes were uniquely present in *G*. *max*, while H5-19 haplotypes were restricted to *G*. *soja*. CC- was specifically present in H1 haplotype and its accessions accounted for 70.7% (335 accessions) of 474 examined accessions, representing the biggest haplotype group. The rest haplotypes had less than 2.4% haplotype frequency except for H3 with 15.4% (73 of 474 accessions). Consistent with our earlier finding, H1-accessions contained 2.5% higher oil content (*p* < 0.001) and 2.2% lower protein content (*p* < 0.001) than those in accessions carrying H2-H4, and much higher oil content (8.25%, *p* < 0.001) and lower protein (4.14%, *p* < 0.001) than all testing *G*. *soja* accessions (H5-H19) (**Fig 4B**). H1 and H2 haplotypes differed only at the CC indel while H1 contained 3.1% higher in oil (*p* < 0.05) and 3.4% lower protein content than in H2 haplotypes, further verifying that CC deletion is the major cause of the observed differences in both traits. In contrast, H3 accessions with 2.5% lower oil content (*p* < 0.001) contained 2.2% higher seed protein (*p* < 0.001) than H1. H1 and H3, collectively representing 98.8% of *G*. *max* accessions in the association panel, were two major haplotypes of *GmSWEET39* that contributed to the phenotypic variations in oil and protein in *G*. *max*. However, none of the DNA variants such as CC indel or haplotype variation of *GmSWEET39* accounts for the dramatic differences in protein and oil between *G*. *soja* and *G*. *max*. This finding was consistent to the above allelic analysis, suggesting that additional QTLs such as QTL on chr20 might contribute to the observed differences in protein and oil content between *G*. *soja* and *G*. *max* accessions.

We also mapped all haplotypes to their geographic origins aimed to understand the use of haplotypes in soybean improvement (**Fig 4C**). Overall, haplotypes (H5-H19) from wild soybean (*G*. *soja*) were restricted to East Asia where wild soybeans originated [21]. In contrast, haplotypes (H1-H4) from cultivated soybean (*G*. *max*) had a wider geographic distribution worldwide (**Fig 4C**). Of the four *G*. *max* haplotypes, H1-carrying accessions with high oil (**Fig 3B**) were widely distributed with 75.52% of all accessions (253) found in Asia followed by 16.42% (55) in North America (**Fig 4D**). It is important to note that the H1 haplotype was nearly fixed in the United States as 95.92% (47 of 49) of US-bred cultivars in this study carried the H1 allele. To further confirm, we examined CC- allele in 75 historically important landrace and milestone cultivars representing North American soybean breeding history from 1907 to 2002. These cultivars belonged to a wide range of maturity groups (MG) from MG 0 to MG VIII [22]. The CC- allele of *GmSWEET39* was present in all of the 75 historical cultivars including the 22 founder cultivars, which have been used in breeding soybean cultivars at varying frequencies (1–44) during the 95 years of North American soybean breeding (**Fig 4E, S4 Table**). These results strongly indicated that the CC- allele of *GmSWEET39* was a preferable allele that has been maintained in modern breeding programs and has been extensively used for soybean improvement worldwide, likely for oil improvement. In contrast, the accessions carrying H3, which contained medium levels of seed oil (16.3%) and protein (45.4%), were mainly from Asia (**Fig 4B–4D**). H3 contained the accessions that have been used for breeding high protein varieties, such as PI407788A from South Korea [23] and Enrei from Japan [24].

## Discussion

### Discovering a major protein and oil QTL gene and its underlying causative alleles using an integrative approach

Soybean genetic studies mapped many important trait QTLs into large confident intervals generally spanning multi-Mb genomic regions in the past decades. It is still a challenge to identify causative QTL genes and DNA variants/alleles for complex traits within such large DNA regions. The recent availability of genome and transcriptome sequencing data of many diverse soybean accessions and tissues provide an opportunity to dissect those large QTL regions at a single nucleotide and gene level to discover their causative QTL genes and alleles. In this study, having fine-mapped a major protein and oil QTL to a 4-Mb region on chr15 using a bi-parental RIL linkage mapping based on SoySNP50K Chip genotyping data, we effectively pinpointed its QTL causative gene and allele to a sugar transporter gene, *GmSWEET39*, and a 2-bp CC deletion through an association analysis with almost all SNPs and Indels in the 4-Mb region of 631 diverse soybean accessions. The major protein and oil QTLs have been repeatedly mapped to the genomic region on chr15 and extensively investigated [4, 8–10, 12, 25–27] in an attempt to identify their causative genes and alleles because of their high effect on protein and oil content and significance in soybean agriculture since it was first identified in the 1990s [10]. Thus, integration of high confident bi-parental genetic linkage mapping with the high-resolution association analysis of the large panel of diverse wild and domesticated soybean accessions should also offer an effective approach/platform to discover causative genes and alleles underlying other complex traits in soybean and enhance translation from phenotype- and marker-based breeding into causative gene/allele based precision breeding.

### Agronomic impacts of the pleiotropic *GmSWEET39* on soybean seed improvement

Previous genetic studies mapped both protein and oil QTLs to the 4.0 Mb region on chr15 [8–10, 27], whereas, whether these traits were controlled by one gene was unknown. Here, we revealed that the complex and highly correlated protein and oil traits were controlled by a single gene *GmSWEET39* with two major types of alleles carrying CC- and CC+ that were highly associated with high oil and high protein, respectively. Differing from the two contemporary studies locating *GmSWEET39* using genetic sweep-inferred approach followed by functional validation [28, 29], we identified *GmSWEET39* and its causative allele (CC Indel) underlying the long-pursued chr15 QTL using the classic genetics and high-resolution GWAS followed by uncovering its dual role for oil and protein improvement. Uncovering the causative gene and alleles for the long-pursued chr15 QTL and the dual functions of the *GmSWEET39* alleles allows us to associate it tightly with soybean decades’ breeding practices and highlight its importance in increasing oil or protein during soybean domestication and improvement. Its agricultural importance was evidenced by the fixation of CC- allele in the US cultivars during the 90-year soybean breeding and its extensive uses in soybean improvement worldwide. Our study demonstrated that the H1 and H3 account for almost all variation of *GmSWEET39* (98.8%) in cultivated soybean and contribute to high oil and high protein respectively. It fits the pleiotropic role of chr15 QTL on seed protein and oil content as revealed in our and previous studies [8–10, 27]. It is likely that different selection pressures on H1 (CC-) and H3 (CC+), based on distinct needs for high oil, high protein soybean cultivars, consequently affected their allelic frequencies in soybean population.

The allelic analysis revealed that *GmSWEET39* experiences two different paths of selection, CC- allele for high oil during domestication and improvement while CC+ allele for high protein during soybean improvement, which was rarely reported in previous studies. However, artificial selection for CC- allele during domestication and improvement, in part, accounted for the decreasing trend of protein content across the transition of wild-landrace-cultivar due to its negative pleotropic effect on protein content (**Fig 3B–3D**). In turn, recruiting its counterpart allele (CC+) would offer an opportunity to reverse the general decline of protein content in CC- modern cultivars. For example, Brzostowski and Diers [23] introgressed a high-protein allele of chr15 QTL in PI407788A into two elite cultivars and showed that lines carrying chr15 QTL from PI407788A contained higher seed protein without significant yield drag compared to the recurrent parents. The Japanese cultivar Enrei contains high seed protein and has been used for breeding high-protein cultivars for soy food production to meet Japanese diet need [24]. Our result show that PI407788A and Enrei carried H3 haplotype. Therefore, H3 haplotype is an important allele that still undergoes ongoing selection at the present-day breeding for high protein content, which is in a great need of animal feed and plant-protein based diets worldwide [3]. Biased presence of H3 haplotype in cultivated soybeans suggest the haplotype likely offers additional beneficial traits to farmers than the other two haplotypes (H2, H4) if it is not caused by a biased population selection. Identification of the soybean accessions containing diverse *GmSWEET39* haplotypes, knowledge on selection, and distribution of the major haplotypes would allow breeders to effectively select and use appropriate haplotypes for target-based seed improvement.

It is known that oil and protein content are complex traits controlled by many genes [7]. Oil and protein content in soybean seeds could be the accumulative or epistatic effects of these QTL genes [30]. Other than *GmSWEET39*, other major QTLs controlling oil and protein content such as *cqPro-20* on chr20 (also identified in our study) and *qOil_05* on chr5 [9, 12] may also contribute to the total phenotypic variation. We observed that oil content increase in CC-accessions over the course of soybean domestication and improvement, and protein increase in CC+ accessions during soybean improvement. In addition, higher oil and protein differences were detected between CC+ and CC- accessions of modern cultivar than of wild soybean and landrace. The increased and augmented difference likely attribute to continuous and differential integration of additional protein and oil QTLs for higher oil and protein respectively during soybean domestication and improvement. Uncovering related QTL genes and their causative DNA variants will provide a more comprehensive insight into the complex genetic basis underlying the oil and protein variation in soybean and enable us to develop effective strategies for precisely improving oil, protein or both in soybean.

### *GmSWEET39* likely functions as a seed coat-specific sugar transporter for seed development

It has been well demonstrated that SWEET proteins function in transporting sugar from source tissues to sink tissues [13, 16, 31]. Developing seed is one of the major sink organs. In seeds, cells have been differentiated into distinct tissues with diverse functions over seed development. Seed coat including outer integument and parenchyma is located at the interface between maternal seed tissues and the filial embryo, and it functions as the “transfer station” to facilitate the movement of unloaded sugar from maternal tissues to the embryo and cotyledon [18, 32]. Evidence have demonstrated that this transporting process is mainly mediated by tissue-specific SWEET proteins in the interface tissues and those proteins are essential for seed filling. For example, in *Arabidopsis*, AtSWEET11, -12, -15 is located to the cell membrane of the seed coat [13]. The *sweet11;12;15* triple *Arabidopsis* mutant is defective in sugar delivery from seed coat to developing embryo, which consequently results in reduced starch and oil content and retarded embryo development [13]. Here, we demonstrated predominant expression of *GmSWEET39* in outer integument and parenchyma of seed coat tissues at the early maturation stage. The developmental expression of *GmSWEET39* was coincident with the stage when protein and lipid begin to accumulate in seeds [33]. It should also be noted that, at the early maturation stage, *GmSWEET39* exhibited decreasing gradients in expression in three connected layers of tissues, from the parenchyma (seed coat) to abaxial epidermis (cotyledon) and to aleurone (**Fig 2J** and **2K**), which was coincident with the possible path of sugar flux from maternal seed coat to filial cotyledon [13, 34]. Interestingly, we observed that the truncated GmSWEET39 (CC-) had a higher level of oil content, as evidenced by the H1 vs H2 comparison (**Fig 4A** and **4B**) and supported by recent reports that overexpression of the truncated GmSWEET39 increased oil in seeds [28, 29]. Previous studies have shown that replacing or removing the C-terminal peptide of sugar transporters significantly increased the transport activity by increasing the affinity for the substrates [35, 36]. GmSWEET39 has ability to transport sugar [29], and our protein structural analysis predicted that C-terminal truncation changed its cytoplasmic domain structure. It will be interesting to experimentally test if C terminus truncated GmSWEET39 can facilitate the sugar transport across the membrane. In addition, it was observed that expression of *GmSWEET39* positively correlated with oil content [28], suggesting that expression variation of *GmSWEET39* potentially also contribute to oil difference in soybean population. Taken together, our study suggests that GmSWEET39 is a highly-specialized protein in transporting sugar from the maternal seed coat to the filial cotyledon over course of seed development.

SWEET proteins are encoded by a large and ancient gene family in divergent plant species, and associated with diverse plant physiological and developmental processes such as disease infection, pollen growth and seed development [13, 31, 37, 38]. Patil et. al [14] showed that soybean contains 52 *GmSWEET* genes that have been clustered into distinct subfamilies. Diverse expression patterns have been observed for those soybean genes, suggesting that *GmSWEET* gene family have been sub-functionalized with distinct expression patterns to meet diverse needs of sugar transporting for plant growth and response to environmental signals [14]. Interestingly, *GmSWEET39* and its orthologue *AtSWEET15* were both predominantly expressed in seed coat tissues and functioned in regulating oil content [13, 28]. Arabidopsis and soybean have been diverged from each other for 90 million years [39]. The conservation of their gene expression pattern and function suggested that both *SWEET* genes preserved an ancient and important function in specifically transporting sugar from seed coat to filial embryo and cotyledon to support seed development and storage reserve production. Interestingly, *GmSWEET15* was identified by its specific expression in the seed endosperm, and silencing of it decreased 60–70% sucrose and resulted in severe seed abortion [31]. The orthologous pair, GmSWEET39 and AtSWEET15, were clustered with *GmSWEET15* and *AtSWEET11* and *12* in the same subfamily [14]. This observation raises a possibility that plants have evolved an ancient *SWEET* subfamily to specifically express in those interface tissues to facilitate transporting sugar from maternal tissues to filial cotyledon to support seed development and seed filling. It will be interesting to examine expression patterns and functions of the other *SWEET* genes in the subfamily to validate the hypothesis.

## Experimental procedures

### Plant materials

A RIL population consisting of 300 lines and a natural population containing 631 diverse soybean accessions were used in this study. This F2:5 RIL population was derived from a genetic cross between soybean (*G*. *max*) cv. Williams82 and wild soybean PI479752 (*G*. *soja*). The parental lines and RILs were planted at the USDA-ARS farms in Beltsville, Maryland, in 2012 and 2015, respectively, each year with two replications in a randomized block design. The seeds per plant were bulk harvested and air-dried. Seed protein and oil content were quantified using near-infrared reflectance (NIR) spectroscopy DA 7250 (Perten Instruments, Sweden) at the USDA-ARS Soybean Genomics and Improvement Laboratory at Beltsville, Maryland. The natural soybean population contained 510 *G*. *max* and 121 *G*. *soja* accessions that were obtained from the U.S. National Plant Germplasm System (https://npgsweb.ars-grin.gov/). The phenotypic data including seed oil and protein content were retrieved and from GRIN (https://www.ars-grin.gov/) and used for association study. No difference was observed for detecting the two major oil and protein QTLs on chr15 and chr20 by comparing adjusted and non-adjusted raw phenotypic data, which eliminated the concern of adverse effect of GRIN-sourced seed phenotypic data (multiple field locations across multiple years) on association power [40]. The soybean accessions used in this study and related information were provided in **S1 Table.**

### DNA extraction and genotyping

DNA extraction was conducted using the CTAB method with minor modifications [41]. The DNA was extracted from fully expanded leaves collected from the RILs. Qualified DNA samples were used for genotyping using the SoySNP50K iSelect SNP BeadChip and the SNPs were determined as previously described [42].

### Generation of genome-wide DNA variants at single-nucleotide resolution

The DNA variants used for association analysis were from three sources including the re-sequencing of *G*. *soja* soybean accessions, SoySNP50K BeadChip [42], and the archived sequencing reads deposited at the public data repository NCBI SRA (https://www.ncbi.nlm.nih.gov/sra). A total of 91 diverse *G*. *soja*, representing over 90% diversity of the wild soybean collection in the US Soybean Collection, were re-sequenced using the Illumina NextSeq 500 sequencer. The sequencing reads from the *G*. *soja* collection and NCBI SRA were processed and aligned to the soybean reference genome *Wm82*.*a2*.*v1* that was downloaded from Phytozome (https://phytozome.jgi.doe.gov) using BWA [43]. The UnifiedGenotyper of the GATKs pipeline [44] was used for the variant (SNP and indel) calling as previously described [44]. The qualified variants from the calling met the following criteria: read depth ≥ 5 & quality ≥ 50 & ≤ 2 SNPs in a 10-bp window allowed. Indel variants were converted to SNP-type variants compatible with association analysis using TASSEL5 [45]. Read alignments were visualized using the Integrative Genomics Viewer [46].

### Linkage and association analysis

The linkage map was constructed as previously described [47]. QTLs were detected by the composite interval mapping procedure of the Windows QTL Cartographer v2.5 [48] with 2.5 as the logarithm of odds ratio (LOD) threshold for detecting a QTL. Association analysis was performed using the mixed model at TASSEL5, using the merged SNPs from three types of DNA variant sources described above. Variants (SNPs and indels) with a minor frequency of ≥ 0.05 on chr15 in the population were used for association analysis. The first five principal components and Kinship matrix calculated using TASSEL5 were included for analysis. We empirically used *P* = 1e-5 as the threshold to determine significant associations. Student’s t-test was carried out to determine the difference significance.

### Tissue-specific expression analysis

Detailed tissue-specific expression patterns were retrieved from Goldberg-Harada Soybean RNA-Seq data (http://seedgenenetwork.net/) derived from developing soybean seeds at different stages and different soybean tissues which were dissected with Laser Capture Microdissection (LCM).

### Genetic diversity analysis

All variant data at the *GmSWEET39* locus were used for its genetic diversity analysis. Data were split into two populations by *G*. *max* and *G*. *soja*. Genomic differentiation values including the fixation index (*Fst*), nucleotide diversity (π), and Tajima’s *D* for *G*. *max* and *G*. *soja* were calculated for 10-kb windows with a 5-kb step using VCFtools [49]. Haplotype analysis was conducted based on the pattern of variants in the region. Haplotypes must contain at least 2 accessions with clear variants at each site. Pedigree data for the 75 milestone and landrace cultivars were obtained from SoyBase (https://www.soybase.org/).

## Supporting information

S1 Fig Distribution of oil and protein content in RILs and the major QTLs. **A** Phenotypic distribution of protein and oil. **B** Correlation between oil content and protein content. **C** Two major QTLs on chr15 and chr 20 were identified using linkage mapping.
(TIF)

S2 Fig Candidate genes within the LD block (A) and the expression patterns in different tissues and maturing seeds at different developing stages (B).
(TIF)

S1 Table Information about the soybean accessions used in association analysis. (PDF)

S2 Table The most significantly associated DNA variants for oil and protein. (PDF)

S3 Table Haplotypes of the accessions identified in this study. (PDF)

S4 Table Information of the 75 landrace and milestone lines used in this study. (PDF)
