# Supplementary figures and images for: Selection of GmSWEET39 for oil and protein improvement in soybean
Source: PLoS Genet. 2020 Nov 11;16(11):e1009114. doi: 10.1371/journal.pgen.1009114 (PMC7721174; doi:10.1371/journal.pgen.1009114)

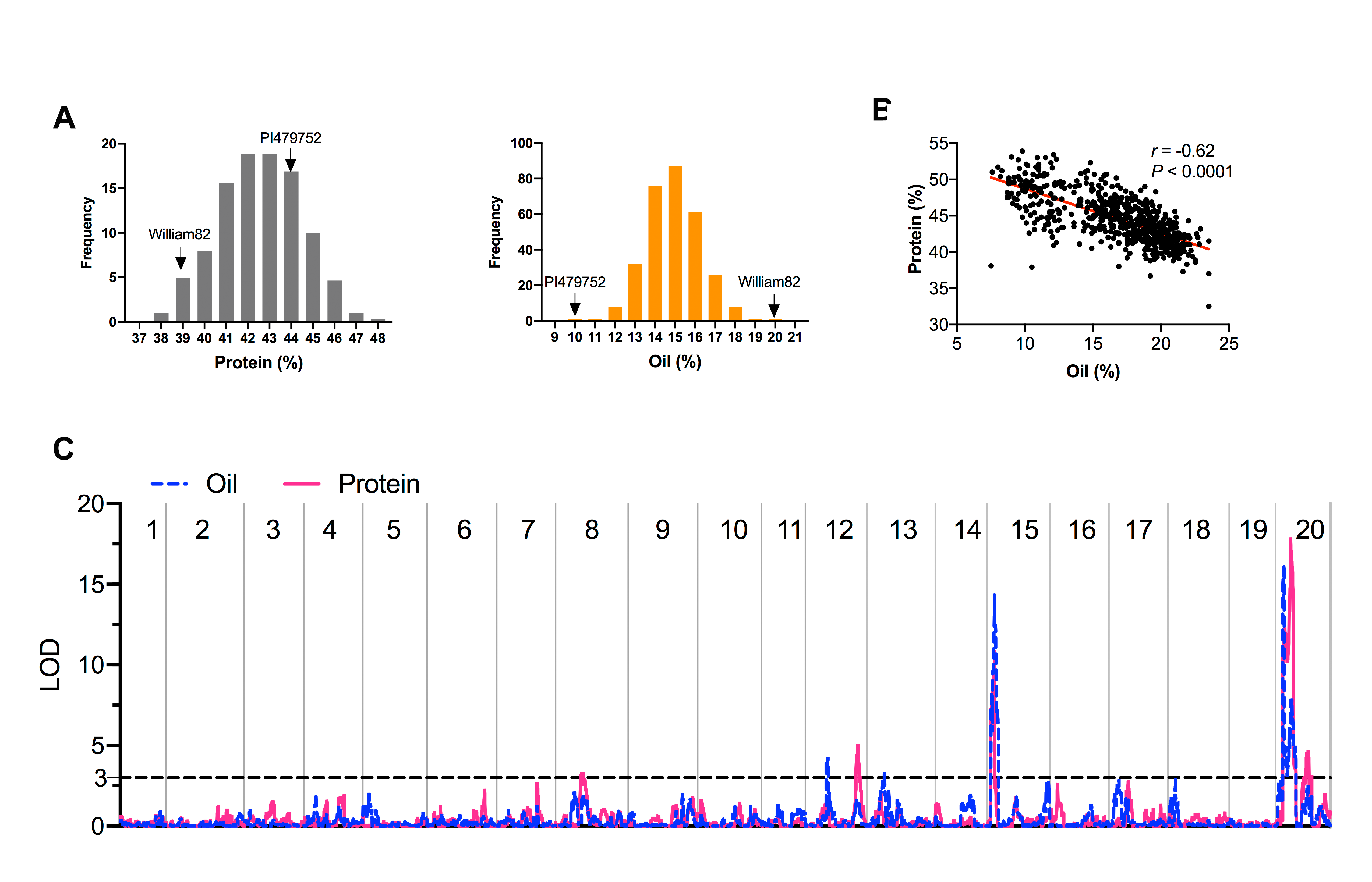

Supplement: S1 Fig — A Phenotypic distribution of protein and oil. B Correlation between oil content and protein content. C Two major QTLs on chr15 and chr 20 were identified using linkage mapping. (TIF) [file pgen.1009114.s001.tif]

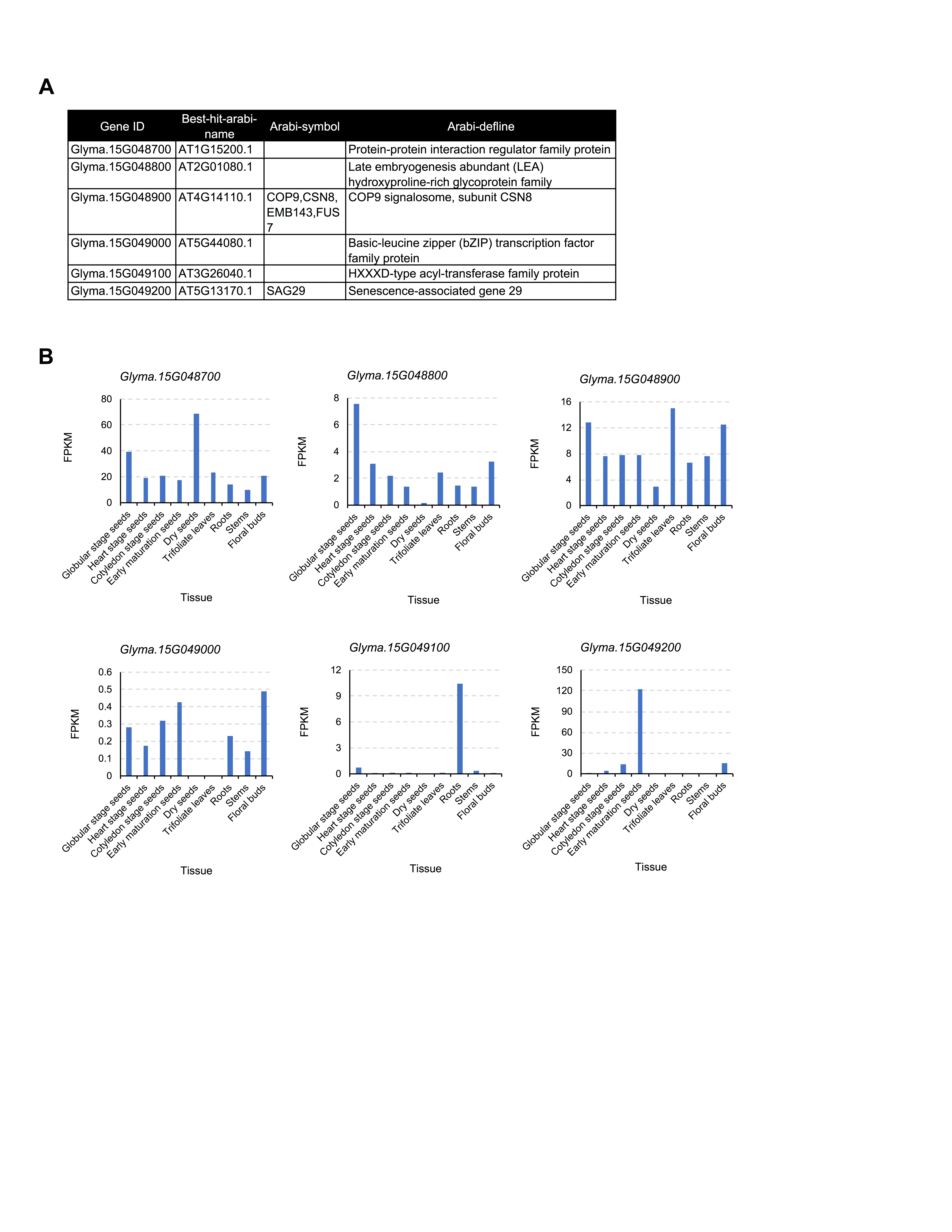

Supplement: S2 Fig — Candidate genes within the LD block (A) and the expression patterns in different tissues and maturing seeds at different developing stages (B). (TIF) [file pgen.1009114.s002.tif]
