# Supplementary material for: Selection of GmSWEET39 for oil and protein improvement in soybean
Source: PLoS Genet. 2020 Nov 11;16(11):e1009114. doi: 10.1371/journal.pgen.1009114 (PMC7721174; doi:10.1371/journal.pgen.1009114)
Supplement: S1 Table — (PDF) [file pgen.1009114.s003.pdf]

S1 Table Information of the accessions used in association analysis

PI	CommonName	Species	MATGROUP	Country	Origin_State_Province	latitude	longitude	OIL (%)	PROTEIN (%)
FC29333	Laredo	Glycine max	III					20.4	40.9
FC31697		Glycine max	IV	Costa Rica		9.91	-84.2	20.4	40.8
FC31721		Glycine max	VI					16.5	46.5
FC33243	Anderson	Glycine max	IV	Unknown	Iowa	41.56	-93.5	21.4	39.8
PI101404A		Glycine soja	II	China	Heilongjiang	46.2	126.07	8.8	49.4
PI103088	Ming Chuan	Glycine max	III	China	Henan	34.74	113.66	17.9	47.1
PI123440	No. 2	Glycine max	VI	Myanmar		19.71	95.37	15.4	47.9
PI153231	B-63	Glycine max	III					19.9	42
PI153262	Roumanie	Glycine max	0	Belgium	Belgium	40.38	116.39	19.1	41.65
PI154189	No. 57	Glycine max	0	Netherlands		52.32	4.89	18.6	41.6
PI157421	Ebony	Glycine max	III	Korea, South	Korea	35.87	128.68	16.9	45.4
PI159925	Glycine H	Glycine max	VIII	Peru	Lima	-12.05	-77.04	15.8	45.5
PI165563	Bhart	Glycine max	VII	India	Uttar Pradesh	29.65	79.78	11.1	50.55
PI165675	Nanking 332	Glycine max	VII	China	Jiangsu	32.06	118.85	17	46.8
PI166105	Bhart	Glycine max	VII	India	Uttar Pradesh	30.25	79.33	14.1	47
PI171428	Large Yellow Soyb	Glycine max	IV	China	Beijing	39.76	116.54	19.1	43.1
PI171451	Kosamame	Glycine max	VII	Japan	Kanagawa	35.59	139.35	15.45	47.15
PI179935	Bhart	Glycine max	VII	India	Himachal Prad	31.88	77.39	16.7	42.7
PI180501	Strain No. 18	Glycine max	0	Germany		52.51	13.41	21.3	39.1
PI189873	Miko Saumon	Glycine max	0	France		48.83	2.22	17.5	44.65
PI196166	No. 2296	Glycine max	V	Korea, South	Korea	35.87	128.68	18.8	44.25
PI196175	Yu tae	Glycine max	V	Korea, South	Korea	35.87	128.68	14.75	52.25
PI200508	Natsu Daizu	Glycine max	I	Japan	Shikoku	33.74	133.66	17.5	39.2
PI209332	No. 4	Glycine max	IV	Japan	Hokkaido	43.46	142.61	17.5	41.5
PI209333	No. 3	Glycine max	VI	Japan	Hokkaido	43.46	142.61	15.4	45.25
PI209334	No. 9	Glycine max	III	Japan	Hokkaido	43.46	142.61	18.1	41.9
PI232992	Kono-Kuradaizu	Glycine max	III	Japan	Saga	33.28	130.28	17.5	41.1
PI240664	Bilomi No. 3	Glycine max	X	Philippines	Luzon	15	121.27	21.1	44.8
PI243541	Shakujo	Glycine max	IV	Japan	Akita	39.57	140.42	16.6	42.6
PI248515	White Hilum Iwat	Glycine max	IV	Japan	Northern part o	36.12	138.12	17.4	40.3
PI253658B	No. 9	Glycine max	I	China	China	40.38	116.39	23.5	36.95
PI253661B	No. 12	Glycine max	III	China		40.38	116.39	21	45.6
PI266806C	No. 4	Glycine max	II	China	Hebei	39.94	119.59	20.2	43.5
PI283327	Pingtung Pearl	Glycine max	V	Taiwan	Taiwan	24.73	121.08	15.6	48.8
PI291294		Glycine max	I	China	Heilongjiang	46.86	126.85	18.5	45.7
PI291309D		Glycine max	II	China	Heilongjiang	45.75	126.65	13.9	46.4
PI291310C		Glycine max	II	China	Heilongjiang	45.75	126.65	18.1	47.2
PI297505	Czi ti No. 5	Glycine max	I	China		40.38	116.39	21.1	44
PI297520	Iregi Universal	Glycine max	0	Hungary		47.49	19.09	20.5	40.75
PI297543	Peking	Glycine max	II	China	North	46.86	126.85	18.6	44.6
PI317334B	(Kitamishiro)	Glycine max	II	Japan	Hokkaido	43.46	142.61	18.3	42.1
PI317336	Shinsei	Glycine max	0	Japan	Hokkaido	43.46	142.61	18.8	39.6
PI323576	H 67-27	Glycine max	IX	India	Uttar Pradesh	29.77	79.77	14.4	43.4

PI324924	Rhosa	Glycine max	V	South Africa		-29.3	26.12	21	39.5
PI339734		Glycine max	IV	Korea, South	Kangwon	37.67	128.62	10.9	50.4
PI339871A		Glycine soja	V	Korea, South	Cheju	33.42	126.5	11.7	46.9
PI342434		Glycine max	V	Japan	Iwate	39.48	141.32	10.1	52.1
PI342619A		Glycine max	0	Russian Feder	Primorye	45	135	14.9	48.65
PI342622A		Glycine soja	I	Russian Feder	Primorye	45	135	8.8	47.75
PI361066B	(F. 56-17)	Glycine max	I	Romania		44.42	26.11	17.8	44.3
PI361070	Faur	Glycine max	0	Romania		44.42	26.11	19.5	39
PI361080	Kormovaia 15	Glycine max	II	Russian Federation		52.98	127.36	15.9	46.5
PI361087	Medias 23	Glycine max	I	Romania		44.42	26.11	18.1	43
PI361093	Novosadska Br. 1	Glycine max	I	Serbia		44.02	21.15	17.8	45.6
PI366120		Glycine soja	IV	Japan	Akita	39.53	140.38	8.95	53.05
PI366122		Glycine soja	IV	Japan	Fukushima	37.46	139.84	8.8	49.9
PI366123		Glycine soja	IV	Japan	Iwate	39.7	141.25	8	51.7
PI366124		Glycine soja	V	Japan	Kagawa	34.23	133.78	9.5	48
PI372403B	(Caloria)	Glycine max	0	Austria		48.2	16.38	19.4	41.7
PI372418	Novosadska Br. 4	Glycine max	I	Serbia		44.02	21.15	17.7	42.5
PI374207	N-9	Glycine max	X	India	Madhya Prades	21.59	78.98	12.2	44.3
PI378658	Dnepropetrovsk 1	Glycine max	0	Ukraine	Dnipropetrovsk	48.45	35	20	41
PI378663	Habarovskaja II	Glycine max	I	Russian Federation		52.98	127.36	16.5	44.5
PI378680E	(VNIIMK 9186)	Glycine max	I	Russian Federation		52.98	127.36	19.4	43.6
PI378684A		Glycine soja	VI	Japan	Saitama	36.03	139.53	8.8	50.2
PI378690		Glycine soja	VII	Japan	Fukuoka	33.2	130.37	10.8	51.7
PI378692		Glycine soja	IV	Japan	Iwate	39.7	141.2	9.2	50.3
PI378696A		Glycine soja	VI	Japan	Shimane	35.12	132.49	12.7	43.4
PI378696B		Glycine soja	VI	Japan	Shimane	35.12	132.49	11.9	44.9
PI378697A		Glycine soja	V	Japan	Aomori	40.6	140.47	10.7	44
PI378698		Glycine soja	VI	Japan	Yamanashi	35.45	138.85	9.6	50
PI378699A		Glycine soja	VII	Japan	Tokyo	35.73	139.57	12.4	43
PI378702		Glycine soja	IV	Japan	Iwate	39.7	141.2	8.3	51.2
PI379618	TC 1	Glycine max	V	Taiwan	Taiwan	24.73	121.08	18.05	43.6
PI385942	Enrei	Glycine max	IV	Japan	Nagano	36.7	137.86	15.1	46.4
PI391577	Ch'a ye sheng tou	Glycine max	II	China	Jilin	44.23	126	14.1	46.4
PI391583	Jilin No. 10	Glycine max	II	China	Jilin	44.23	126	20	43.7
PI393551		Glycine soja	X	Taiwan	Taiwan	24.73	121.08	9.2	47.4
PI398296	KAS 173-3	Glycine max	II	Korea, South	Kyonggi	37.14	127.07	18.35	44.95
PI398593	KAS 390-4	Glycine max	V	Korea, South	Chungchong Pu	37	128.17	11.45	53
PI398595	KAS 390-5	Glycine max	V	Korea, South	Chungchong Pu	37	128.17	12.25	52.75
PI398610	KAS 390-8	Glycine max	V	Korea, South	Chungchong Pu	37	128.17	14.05	50.75
PI398614	KAS 390-9	Glycine max	V	Korea, South	Chungchong Pu	37	128.17	14.35	51
PI398633	KAS 390-17-2	Glycine max	V	Korea, South	Chungchong Pu	37	128.17	13.95	49.2
PI398881	KLS 126-1	Glycine max	III	Korea, South	Kyonggi	37	127	20.45	42.85
PI398965	KLS 628-1	Glycine max	IV	Korea, South	Cholla Nam	34.5	126.38	18.6	44.2
PI399043	KLS 903	Glycine max	III	Korea, South	Cheju	33.4	126.55	18.3	44
PI404166	Krasnoarmejskaja	Glycine max	III	China		40.38	116.39	15.5	43.1
PI404182	Sin i tu li rau	Glycine max	III	China	China	40.38	116.39	16.7	46.2
PI404187	Suj nii hun mao ju	Glycine max	II	China		40.38	116.39	16.8	46.3

PI404188A	Huaj an si er dian	Glycine max	II	China	N/A	40.38	116.39	19.3	43
PI404198B	(Sun huan do)	Glycine max	IV	China		40.38	116.39	14.3	42.9
PI407027	RB 1072	Glycine soja	V	Japan	Akita	39.57	140.42	11.6	50.7
PI407038	RB 1072	Glycine soja	V	Japan	Akita	39.7	140.73	11.4	50.8
PI407042	RB 1072	Glycine soja	V	Japan	Akita	39.57	140.42	11.2	44.8
PI407052	RB 1072	Glycine soja	V	Japan	Iwate	39.72	141.14	10.1	49
PI407077	RB 1072	Glycine soja	VII	Japan	Aichi	34.94	137.24	11.7	44.6
PI407083	RB 1072	Glycine soja	VI	Japan	Aichi	34.94	137.24	9.5	50.4
PI407096	RB 1072	Glycine soja	VII	Japan	Hyogo	34.92	135.23	10.7	47.9
PI407157	RB 1072	Glycine soja	VI	Japan	Chiba	35.6	140.12	10.4	49.2
PI407162	K1-D	Glycine soja	IV	Korea, South	Kyonggi	37.28	127.11	10.1	49.75
PI407170	K2-E	Glycine soja	V	Korea, South	Kyonggi	37.28	127.12	11.5	50.7
PI407171	K2-F	Glycine soja	IV	Korea, South	Kyonggi	37.28	127.12	8.7	48.2
PI407175	K4-B	Glycine soja	IV	Korea, South	Kyonggi	37.2	127.44	9.8	48.5
PI407183	K8-B	Glycine soja	V	Korea, South	Kyonggi	37.74	127.43	8.8	47.6
PI407184	K29	Glycine soja	IV	Korea, South	Kyonggi	37.24	127.02	9.1	49.6
PI407190	K56-C	Glycine soja	V	Korea, South	Kyonggi	37.21	126.99	10.3	49.4
PI407195	K12-A	Glycine soja	IV	Korea, South	Kangwon	37.68	127.88	10.45	48.9
PI407202	K15	Glycine soja	V	Korea, South	Kangwon	37.5	127.98	10.1	45.7
PI407214	K23-A	Glycine soja	V	Korea, South	Chungchong Pu	37.05	127.95	12.7	49
PI407220	K25-B	Glycine soja	V	Korea, South	Chungchong Pu	36.95	127.74	12.1	47.4
PI407229	K47-A	Glycine soja	V	Korea, South	Chungchong Na	36.5	127.24	11.9	42.8
PI407239	K55	Glycine soja	V	Korea, South	Chungchong Na	36.76	127.16	11.8	45.4
PI407241	K35-B	Glycine soja	V	Korea, South	Kyongsang Puk	35.6	128.75	11.7	46.8
PI407243	K36-B	Glycine soja	VI	Korea, South	Kyongsang Puk	35.61	128.75	9.6	46.1
PI407248	K37-D	Glycine soja	V	Korea, South	Kyongsang Puk	35.68	128.75	11	52.1
PI407275	K101-A	Glycine soja	IV	Korea, South	Kyonggi	37.43	126.99	10.65	48.95
PI407285	Yasei Daizu	Glycine soja	VII	Japan	Kanagawa	35.59	139.35	9.5	52.7
PI407286	Tsuru Mame	Glycine soja	VI	Japan	Kanagawa	35.59	139.35	11	48.5
PI407288		Glycine soja	II	China	Jilin	43.51	124.81	9.15	49.55
PI407300		Glycine soja	V	China	Jiangsu	32.06	118.85	10.7	47.9
PI407303		Glycine soja	VI	China	Jiangsu	32.06	118.85	10.6	48.9
PI407307		Glycine soja	VI	China	Shanghai	31.15	121.8	12.5	45.6
PI407308		Glycine soja	V	Korea, South	Kyonggi	37.28	127.02	10.6	50.7
PI407315		Glycine soja	V	Korea, South	Chungchong Pu	36.93	128.98	11.4	50.5
PI407318A		Glycine soja	V	Korea, South	Chungchong Pu	36.54	127.83	12.4	49.5
PI407701	Hei long No. 3	Glycine max	I	China		40.38	116.39	20.8	43.6
PI407708A	Feng shou No. 10	Glycine max	0	China	Heilongjiang	46.86	126.85	19.45	43.45
PI407716	Jin nung No. 3	Glycine max	I	China	Jilin	44.23	126	19	46.7
PI407729		Glycine max	IV	China	Beijing	40.38	116.39	16.2	46.9
PI407742	16	Glycine max	V	China	Shaanxi	34.26	108.93	18.35	46.9
PI407788A	ORD 8113	Glycine max	IV	Korea, South	Kyonggi	37.83	127.51	15.1	50.7
PI407801		Glycine max	VI	Korea, South	Kyonggi	37.3	127.63	12.9	48.8
PI407849	KAS 510-1	Glycine max	III	Korea, South	Cholla Puk	35.95	126.96	18.1	43.8
PI407965	KAERI 504-4	Glycine max	V	Korea, South	Cholla Nam	35.23	126.31	14.85	49.25
PI408105A	KAS 633-19	Glycine max	IV	Korea, South	Kyongsang Puk	36.08	129.36	17.6	46.6
PI416751	A-B(D)	Glycine max	I	Japan	Tohoku region	39	141	19.4	44.6

PI416838	Choutan	Glycine max	V	Japan	Tohoku region	39	141	19.9	42.4
PI416890	Gokuwase natsu d	Glycine max	0	Japan	Japan	35.94	139.4	15.5	47.6
PI416937	Houjaku Kuwazu	Glycine max	VI	Japan	Kanto and Tosa	36	139	15.6	47.1
PI416971	Kaifuu gyuumou d	Glycine max	IV	Japan	Kinki	34.7	135.5	16.7	42.6
PI417015	Kawanagare (Iwat	Glycine max	III	Japan	Iwate	39.48	141.32	17	44.1
PI417091	Kuro mame	Glycine max	II	Japan	Kanto and Tosa	36	139	16.4	43.35
PI417215	Ooita Aki Daizu 2	Glycine max	VIII	Japan	Kyushu and Ok	26.54	128.02	19.6	43.6
PI417242	Pekin dai seitou	Glycine max	II	China		40.38	116.39	18.7	44.8
PI417345B	(Shou outou)	Glycine max	IV	China		40.38	116.39	16.4	46.2
PI417381	Tenpoku shirome	Glycine max	0	Japan	Hokkaido	43.46	142.61	18.9	43.5
PI417479	Yougetsu	Glycine max	IV	Japan	Tohoku region	39	141	16.9	44.5
PI417500	Escura A	Glycine max	VIII	Brazil		-14	-46.6	14.9	46.4
PI417529	A38	Glycine max	0	Germany		52.51	13.41	19.1	45.5
PI417581	H-060072	Glycine max	V	United States		38.67	-90.4	19.5	45.7
PI423766	KAS 230-4	Glycine max	IV	Korea, South	Kangwon	37.17	128.5	17.8	42.4
PI423926	Tousan 72	Glycine max	IV	Japan	Nagano	36.7	137.86	16.5	43.4
PI423954	Shirome	Glycine max	0	Japan	Kumamoto	32.88	130.97	15.9	49.5
PI423967	Nabeshima	Glycine max	IX	Japan	Kumamoto	32.88	130.97	16.4	45.6
PI424007	74005	Glycine soja	V	Korea, South	Kyonggi	37.21	126.82	12.9	46.1
PI424014	74012	Glycine soja	V	Korea, South	Kyonggi	37.28	127.02	12.2	49.5
PI424025B	74023	Glycine soja	V	Korea, South	Kyonggi	37.9	127.2	10.9	53
PI424035	74038	Glycine soja	V	Korea, South	Kyonggi	37.9	126.98	11.2	47
PI424045	74060	Glycine soja	V	Korea, South	Kyonggi	37.55	127.26	12	51.6
PI424078	74077	Glycine max	III	Korea, South	Kangwon	37.6	128.44	12.2	47.4
PI424082	74082	Glycine soja	V	Korea, South	Kangwon	37.26	128.42	10.9	48.8
PI424083A	74083	Glycine soja	V	Korea, South	Kangwon	37.23	128.42	11.3	50.5
PI424088	74088	Glycine soja	IV	Korea, South	Chungchong Pu	37.13	128.22	9.95	50.85
PI424096	74148	Glycine soja	V	Korea, South	Chungchong Na	36.37	127.19	7.5	38.1
PI424097	74096	Glycine soja	V	Korea, South	Kyongsang Puk	36.12	128	11	46.6
PI424102A	74101	Glycine soja	V	Korea, South	Kyongsang Puk	36.5	128.15	12.6	49.7
PI424107A	74106	Glycine soja	VI	Korea, South	Kyongsang Puk	36.78	128.59	9	48.1
PI424116	74116	Glycine soja	IV	Korea, South	Kyongsang Puk	36.02	128.97	11.2	48.15
PI424121	74129	Glycine soja	V	Korea, South	Kyongsang Nar	34.97	128.32	11.6	49.2
PI424195A	ISZ-3	Glycine max	0	Hungary		47.49	19.09	20.1	40.6
PI424298	KAS 300-10	Glycine max	IV	Korea, South	Chungchong Na	36.33	127.42	15.1	43.2
PI424391	KAS 521-15	Glycine max	VI	Korea, South	Cholla Puk	35.63	127.25	17.7	46.1
PI424608A	KAS 681-21	Glycine max	IV	Korea, South	Kyongsang Puk	35.83	129.25	15.6	46.1
PI427136	Backchung No.42	Glycine max	III	Korea, South	Kyonggi	37.28	127.12	19.6	42.5
PI430595	58-161	Glycine max	IV	China		40.38	116.39	16.3	43.9
PI436684	Tie-feng 8	Glycine max	III	China	Liaoning	41.2	122.34	19.6	42.3
PI437110A	VIR 244	Glycine max	III	Russian Feder	Jewish Region,	48.6	132.3	14.3	44.1
PI437112A	VIR 249	Glycine max	II	Russian Feder	Jewish Region,	48.6	132.3	15.4	48.2
PI437127A	Imeretinscaja	Glycine max	IV	Georgia		42.31	43.37	17.6	43.9
PI437165A	Toncostebel'naja	Glycine max	I	Russian Feder	Krasnodar	45.1	39.27	14.9	46.6
PI437169B	(VNIISC-4)	Glycine max	II	Russian Feder	Krasnodar	45.1	39.27	22	42.3
PI437240	CSchi 1069	Glycine max	0	Moldova		47	29	19.1	41
PI437321	Dunganscaja 462	Glycine max	III	Russian Feder	Primorye	45	135	16.1	42

PI437376A	Ussurijscaja 308	Glycine max	I	Russian Feder	Primorye	45	135	19.3	43.2
PI437485	VIR 1048	Glycine max	II	Russian Feder	Primorye	45	135	17.9	41.4
PI437500A	VIR 3810	Glycine max	I	Russian Feder	Primorye	45	135	20.2	42.95
PI437505	VIR 3853	Glycine max	II	Russian Feder	Primorye	45	135	17.8	48.1
PI437653	Er-da-li	Glycine max	II	China	China	40.38	116.39	18.5	42.6
PI437654	Er-hej-jan	Glycine max	III	China	China	40.38	116.39	15	45
PI437655	Er-huan-jan	Glycine max	III	China		40.38	116.39	18.15	44.5
PI437662	Gun'-tszu-lin' 658	Glycine max	II	China		40.38	116.39	12.4	45.8
PI437679	Nan-cou	Glycine max	IV	China	China	40.38	116.39	15.2	45.1
PI437685D	(Phun-zhun)	Glycine max	III	China		40.38	116.39	17	44.6
PI437690	Pin-din-guan	Glycine max	III	China		40.38	116.39	14.5	45.5
PI437725	Te-zu-gan	Glycine max	IV	China		40.38	116.39	15.5	43.7
PI437776	VIR 1302	Glycine max	III	China		40.38	116.39	18.05	43.8
PI437788A	VIR 3018	Glycine max	II	China		40.38	116.39	18	43.3
PI437793	VIR 3024	Glycine max	II	China		40.38	116.39	18.9	43
PI437814A	An'da	Glycine max	II	China	Northeast	40.38	116.39	18.3	44.5
PI437838	DV-254	Glycine max	II	Russian Federation		52.98	127.36	20.5	43.9
PI437863A	DV-2841	Glycine max	II	China	Northeast	40.38	116.39	20.3	42.7
PI437944	VIR 569	Glycine max	II	China	Northeast	40.38	116.39	14.9	44.55
PI437991B	VIR 1657	Glycine max	0	China	Northeast	40.38	116.39	20.4	43
PI438019B	VIR 1883	Glycine max	II	China	Northeast	40.38	116.39	18.5	45.7
PI438083	VIR 2506	Glycine max	II	China	Northeast	40.38	116.39	18.8	44
PI438112B	VIR 2623	Glycine max	III	China	Northeast	40.38	116.39	18.3	42.8
PI438230A	VIR 4521	Glycine max	I	China	Northeast	40.38	116.39	18	43.5
PI438239B	VIR 4536	Glycine max	I	China	Northeast	40.38	116.39	16.5	43.6
PI438309	VIR 3017	Glycine max	I	China		40.38	116.39	16.7	45.8
PI438323	Grignon 53-F-3	Glycine max	I	France		48.83	2.22	17.9	41.2
PI438335	SAO 196-C	Glycine max	III	Algeria		26.66	2.14	18.1	43
PI438336	Sao 208	Glycine max	0	Algeria		27.68	1.79	19.5	39.2
PI438347	35S.277	Glycine max	VII	Australia		-37.81	145.45	18.3	44.5
PI438471	Fiskeby III	Glycine max	0	Sweden	Ostergotland	58.4	15.66	20.3	40.3
PI438496B	(Peking)	Glycine max	III	United States		38.67	-90.4	14.6	46.9
PI438496C	(Peking)	Glycine max	IV	United States		38.67	-90.4	14.1	47.3
PI438498	Sable	Glycine max	IV	United States	United States	38.67	-90.4	15.8	43.2
PI438500	Virginia	Glycine max	III	United States		38.67	-90.4	19	42.05
PI445824A	Wolfsthaler	Glycine max	0	Germany		52.51	13.41	18.8	40.3
PI447003A		Glycine soja	0	China	Nei Monggol	42.82	113.29	9.7	49.15
PI458510	Ji Ti No. 1	Glycine max	III	China	Liaoning	41.2	122.34	21.7	40.05
PI458515	Tie Zhugan	Glycine max	IV	China	Shandong	36.6	118.97	17.3	42.4
PI458828	Qun Xuan No.1	Glycine max	II	China	Jilin	44.23	126	20.3	40
PI464890B	(Gong di No. 2019	Glycine soja	I	China	Jilin	44.23	126	9.95	49.55
PI464896	Jou Nong No. 5	Glycine max	I	China	Jilin	44.23	126	20.5	41.95
PI464912	Dan Dou 1	Glycine max	IV	China	Liaoning	40.14	124.39	19.2	42.9
PI464920B	(Jin Dou 33)	Glycine max	III	China	Liaoning	41.2	122.34	20.55	40.6
PI464923	Tie Fen 16	Glycine max	I	China	Liaoning	42.46	124.04	21.9	38.3
PI464927A	LS-005	Glycine soja	0	China	Liaoning	42.46	124.04	10.8	47.7
PI464929A	LS-009	Glycine soja	0	China	Liaoning	40.8	122.78	9.95	48.3

PI464935	81-200002	Glycine soja	VI	China	Jiangsu	32.06	118.85	11.6	44.7
PI467312	Cha-mo-shi-dou	Glycine max	II	China	Jilin	44.23	126	14.9	43.4
PI467343	Yan-nong No. 2	Glycine max	I	China	Jilin	44.23	126	21.15	42.1
PI467347	Zi-hua-cuo-zi	Glycine max	II	China	Jilin	44.23	126	19.1	41.3
PI468400A		Glycine soja	IV	China	Ningxia	37.33	106.04	11.45	43.7
PI468408B	(Qi Huang No. 1)	Glycine max	III	China		40.38	116.39	19.35	42.5
PI468908		Glycine max	0	China	Jilin	44.23	126	13.3	49.1
PI468915		Glycine max	II	China	Liaoning	41.2	122.34	17.6	42.45
PI468916		Glycine soja	III	China	Liaoning	41.2	122.34	10.1	44
PI468918		Glycine soja	III	China	Liaoning	41.2	122.34	10.85	44.45
PI471938	197	Glycine max	V	Nepal	Jumia	28.13	84.14	21	39.4
PI475783B	(Tsing 2)	Glycine max	III	China	Shanxi	37.33	111.83	18.35	44.1
PI475820		Glycine max	II	China	Xinjiang	46.86	83.23	18.3	42.7
PI476352B	(Colnon)	Glycine max	II	Kyrgyzstan	Kirghiz SSR	41.21	74.78	19.5	40.9
PI479735	Silihuang	Glycine max	III	China	Jilin	43.63	126.5	19.6	39.9
PI479746B	GD 50062	Glycine soja	II	China	Jilin	44.23	126	10	48.95
PI479751	GD 50351-1	Glycine soja	III	China	Jilin	44.23	126	10.35	47.7
PI479752	GD 50388-2	Glycine soja	I	China	Jilin	44.23	126	9.4	46.35
PI479768	Long 79-3313-1	Glycine soja	0	China	Heilongjiang	46.86	126.85	10	48.85
PI479769	Long 79-5801	Glycine soja	0	China	Heilongjiang	48.48	127.97	9.05	46.65
PI483252	Doko	Glycine max	IX	Brazil		-14	-50	18.8	41.4
PI483460B		Glycine soja	III	China	Liaoning	41.1	123.25	8.85	47.95
PI483463		Glycine soja	III	China	Shanxi	38.78	113.42	12.25	41.3
PI483465		Glycine soja	V	China	Shaanxi	34.88	110.01	12.5	44.4
PI490766	Dawudou	Glycine max	III	China	Hebei	39.94	119.59	23.5	41.5
PI490768	Rao Shan Gun	Glycine max	III	China	Hebei	39.94	119.59	17.4	43.3
PI495017C	(Beijing da qing d	Glycine max	IV	China	Beijing	40.38	116.39	18.4	43.65
PI495020	Xu dou 2	Glycine max	IV	China	Beijing	40.38	116.39	16.6	44.5
PI497953	I.C. 192	Glycine max	IX	India	Bihar	25.98	85.68	16.3	44.35
PI497964A	I.C. 9461	Glycine max	Unknow	India	Sikkim	27.33	88.54	19.1	42.8
PI497967	PLSO 96	Glycine max	VII	India	Jammu and Ka	33.89	76.66	15.4	43.35
PI504288	S	Glycine max	V	Japan	Iwate	39.67	141	8.2	50.4
PI506933	Kouiku 1	Glycine max	IV	Japan	Kyushu and Ok	26.54	128.02	14.6	43.7
PI506942	Koushurei 235	Glycine max	II	Japan	Tohoku	39	141	21.1	40.7
PI507017	Madara ooha tsu	Glycine max	VII	Japan	Kanto and Tosa	36	139	11.9	52.3
PI507088	Nattou Kotsubu	Glycine max	VI	Japan	Kanto and Tosa	36	139	15	46.9
PI507293B	(Shoukin ou)	Glycine max	III	Japan	Hokuriku	37	137.5	20.25	40.8
PI507354	Tokei 421	Glycine max	I	Japan	Hokkaido	43.46	142.61	19.2	38.8
PI507355	Tokei 423	Glycine max	I	Japan	Hokkaido	43.46	142.61	19.6	39.9
PI507458	Tousan kei BL 521	Glycine max	IV	Japan	Kanto and Tosa	36	139	21.3	42.6
PI507467	Tousan kei F 764	Glycine max	IV	Japan	Kanto and Tosa	36	139	19.1	41.9
PI507471	Tousan kei na 16	Glycine max	III	Japan	Kanto and Tosa	36	139	20	41.65
PI507480	Tousan kei YL 24	Glycine max	IV	Japan	Kanto and Tosa	36	139	17	45.2
PI507595	NIAR 030002	Glycine soja	VI	Japan	Niigata	37.01	138.65	12.1	44.8
PI507609	NIAR 040023	Glycine soja	VI	Japan	Tochigi	36.55	139.73	8.7	47.5
PI507615	NIAR 040012	Glycine soja	VI	Japan	Nagano	36.4	138.25	10.4	45.1
PI507618	NIAR 040015	Glycine soja	V	Japan	Nagano	36.7	137.86	10.3	42.6

PI507619B	NIAR 040016	Glycine soja	VI	Japan	Nagano	36.7	137.86	9.8	44.8
PI507630	NIAR 060002	Glycine soja	VII	Japan	Nara	34.53	135.95	10.6	46.6
PI507638	NIAR 060010	Glycine soja	VI	Japan	Hyogo	34.79	134.85	12	42
PI507641	NIAR 060014	Glycine soja	V	Japan	Hyogo	35.4	134.77	10	46.2
PI507656	NIAR 090011	Glycine soja	VII	Japan	Nagasaki	33	129.5	10.6	48.8
PI507662	NIAR 090019	Glycine soja	VIII	Japan	Kagoshima	31.33	130.93	10.2	51.9
PI507667	NIAR 090024	Glycine soja	VI	Japan	Kumamoto	32.67	130.69	10.7	45.4
PI507681B	(Jangiabad)	Glycine max	II	Uzbekistan	N/A	41.31	69.25	18.4	42.9
PI507752	VIR 8448	Glycine soja	0	Russian Feder	Amur	52.98	127.36	14.9	40.5
PI507757	VIR 8451	Glycine soja	0	Russian Feder	Amur	64.41	144.03	12.8	43.4
PI507761	VIR 8455	Glycine soja	I	Russian Feder	Amur	52.98	127.36	10.7	43.1
PI507830B	VIR 9044	Glycine soja	0	Russian Feder	Amur	52.98	127.36	11	51.7
PI507847	L 598	Glycine soja	II	Russian Feder	Amur	52.98	127.36	11.4	43.1
PI508083	Dassel	Glycine max	0	United States	Minnesota	45.18	-93.36	20.8	41.033
PI508266	Young	Glycine max	VI	United States	North Carolina	35.22	-80.76	19.44	44.16
PI513382	Glenwood	Glycine max	0	United States	Minnesota	45.18	-93.36	20.6	41.5
PI514671	Feng shou No. 7	Glycine max	0	China	Heilongjiang	46.86	126.85	18.8	46.9
PI515961	Pennyrile	Glycine max	IV	United States	Kentucky	38.2	-85.76	19.6	41.7
PI518668	TN 4-86	Glycine max	IV	United States	Tennessee	36.18	-86.81	18.8	43
PI518703	He feng 25	Glycine max	I	China	Heilongjiang	46.83	130.35	20.2	44.5
PI518711	Jilin 21	Glycine max	II	China	Jilin	43.51	124.81	22	44.3
PI518727	Ju huang	Glycine max	VI	China	Guangdong	23.12	113.25	17.3	46.5
PI518750	NS-16	Glycine max	I	Former Serbia	Former Serbia	44.02	21.15	22.7	42
PI518751	NS-20	Glycine max	II	Former Serbia	N/A	44.02	21.15	21.1	43.4
PI522182B	ZYD 253	Glycine soja	I	China	Heilongjiang	48.48	127.97	9.7	49.9
PI522209B	VIR 8493	Glycine soja	II	Russian Feder	Primorye	45	135	12.1	40.9
PI522216	VIR 8500	Glycine soja	III	Russian Feder	Primorye	45	135	10.5	37.9
PI522226	VIR 8511	Glycine soja	0	Russian Feder	Primorye	45	135	9.4	47.2
PI522228	VIR 8514	Glycine soja	I	Russian Feder	Primorye	45	135	12	42.1
PI522235B	VIR 8525	Glycine soja	I	Russian Feder	Primorye	45	135	11.6	44.7
PI525453	Conrad	Glycine max	II	United States	Iowa	41.56	-93.5	21.25	40.4
PI532450	GD50344	Glycine soja	I	China	Jilin	42	126	11.7	44
PI532463B	(He bei No. 1)	Glycine max	III	China	Hebei	39.94	119.59	18.7	45.6
PI533655	Burlison	Glycine max	II	United States	Illinois	39.76	-89.57	19.4	43.3
PI538380	Si Jiao Qi Huang D	Glycine max	IV	China	Hebei	36.81	115.07	18.3	46
PI538386A	1886	Glycine max	III	China	Hebei	39.94	119.59	16.2	45.2
PI540552	Hoyt	Glycine max	II	United States	Ohio	39.94	-83.09	21.4	40.6
PI540556	Jack	Glycine max	II	United States	Illinois	39.76	-89.57	19.7	39.3
PI542044	Kunitz	Glycine max	III	United States	Illinois	39.76	-89.57	20.9	44.1
PI542403	Dawson	Glycine max	0	United States	Minnesota	45.18	-93.36	20.87	39.967
PI54591	No. 31	Glycine max	III	China	Liaoning	41.2	122.34	19.8	41.5
PI54608-1	No. 48	Glycine max	Unknown	China	Liaoning	41.2	122.34	20.3	41
PI54614	No. 54	Glycine max	IV	China	Jilin	43.88	125.32	20.1	44
PI54615-1	No. 55	Glycine max	Unknown	China	Heilongjiang	45.75	126.65	19	44.4
PI547403	L60-246	Glycine max	IV	United States	Illinois	39.76	-89.57	22.3	39.4
PI547409	L62-1251	Glycine max	IV	United States	Illinois	39.76	-89.57	22.5	38.9
PI547460	L64-1083	Glycine max	IV	United States	Illinois	39.76	-89.57	19.1	44.3

PI547612	L74-826	Glycine max	IV	United States	Illinois	39.76	-89.57	21.1	40.7
PI547680	L62-17	Glycine max	II	United States	Illinois	39.76	-89.57	22.9	43.2
PI547690	L63-1212	Glycine max	II	United States	Illinois	39.76	-89.57	22.8	41.1
PI547716	L62-667	Glycine max	II	United States	Illinois	39.76	-89.57	22.2	40.2
PI547831	L83-0215	Glycine soja	II	United States	Illinois	39.76	-89.57	7.6	51
PI548193	T201	Glycine max	IV	United States	Iowa	41.56	-93.5	23.5	32.5
PI548298	A.K. (Harrow)	Glycine max	III	China	China	40.38	116.39	20.2	41.85
PI548311	Capital	Glycine max	0	Canada	Ontario	43.69	-79.4	20.4	42
PI548313	Chestnut	Glycine max	III	Russian Feder	Khabarovsk	48.5	135.13	20.6	39.6
PI548316	Cloud	Glycine max	III	China	Zhejiang	30.27	120.08	18.4	42.1
PI548317	Columbia	Glycine max	III	China	Hebei	39.94	119.59	20.4	40
PI548336	Habaro	Glycine max	I	Russian Feder	Khabarovsk	48.5	135.13	20.8	42
PI548342	Higan	Glycine max	IV	Japan	Tokyo	35.73	139.57	19.3	41.2
PI548348	Illini	Glycine max	III	China	Northeast	40.38	116.39	20.7	41.5
PI548349	Ilsoy	Glycine max	III	Korea, North	Pyongyang	39.04	125.75	18.1	41.5
PI548356	Kanro	Glycine max	II	Korea, North	Pyongyang	39.04	125.75	19.9	41.4
PI548360	Korean	Glycine max	II	Korea, North		39.04	125.75	21.7	41.9
PI548364	Macoupin	Glycine max	IV	Japan	Tokyo	35.73	139.57	21.3	40.2
PI548379	Mandarin (Ottawa	Glycine max	0	China	Heilongjiang	46.86	126.85	19.95	43.6
PI548382	Manitoba Brown	Glycine max	0	China	Liaoning	41.2	122.34	17.85	43.6
PI548383	Mansoy	Glycine max	III	China	Heilongjiang	46.86	126.85	20.7	43.45
PI548391	Mukden	Glycine max	II	China	Liaoning	41.2	122.34	20.5	42.5
PI548400	Patoka	Glycine max	IV	China	Heilongjiang	46.86	126.85	19.85	43.3
PI548406	Richland	Glycine max	II	China	Jilin	44.23	126	20.9	41.5
PI548411	Seneca	Glycine max	II	China	Northeast Chin	40.38	116.39	21.2	39.4
PI548415	Sooty	Glycine max	IV	China	Zhejiang	30.27	120.08	16.9	41.3
PI548417	Soysota	Glycine max	I	Italy	Naples	40.85	14.26	20.95	42.55
PI548445	CNS	Glycine max	VII	China	Jiangsu	32.06	118.85	17.45	47.35
PI548447	Cherokee	Glycine max	VIII	China	Zhejiang	30.27	120.08	14.9	48.4
PI548452	Dixie	Glycine max	V	Korea, North	Pyongyang	39.04	125.75	19.5	42.7
PI548456	Haberlandt	Glycine max	VI	Korea, North	Pyongyang	39.04	125.75	20	41.5
PI548473	Monetta	Glycine max	VII	China	Jiangsu	32.06	118.85	15.9	45.45
PI548474	Nanda	Glycine max	VIII	Korea, North	Hwanghae Puk	38.51	125.76	17.85	41.7
PI548477	Ogden	Glycine max	VI	United States	Tennessee	36.18	-86.81	20	42.6
PI548479	Otootan	Glycine max	VIII	Taiwan	Taiwan	24.73	121.08	14.6	46.1
PI548485	Roanoke	Glycine max	VII	China	Jiangsu	32.06	118.85	19.35	43.5
PI548488	S-100	Glycine max	V	China	Heilongjiang	46.86	126.85	18.95	45.95
PI548490	Tanner	Glycine max	VII	Taiwan	Taiwan	24.73	121.08	16.3	40.7
PI548510	Beeson	Glycine max	II	USA	Indiana	39.78	-86.31	19.2	44.2
PI548511	Beeson 80	Glycine max	II	United States	Indiana	39.78	-86.31	19.3	42.8
PI548512	Century	Glycine max	II	United States	Indiana	39.78	-86.31	20.2	43.6
PI548520	Preston	Glycine max	II	United States	Iowa	41.56	-93.5	21.85	40.95
PI548521	BSR 201	Glycine max	II	United States	Iowa	41.56	-93.5	20.3	41.5
PI548533	Clark	Glycine max	IV	United States	Illinois	39.76	-89.57	21.2	41.1
PI548540	Corsoy	Glycine max	II	United States	Iowa	41.56	-93.5	19.9	42.5
PI548561	Hodgson	Glycine max	I	United States	Minnesota	45.18	-93.36	21	42.2
PI548565	Gnome	Glycine max	II	United States	Ohio	39.94	-83.09	20.5	43.95

PI548571	Harlon	Glycine max	I	Canada	Ontario	43.69	-79.4	21.47	39.986
PI548573	Harosoy	Glycine max	II	Canada	Ontario	43.69	-79.4	21.6	41.2
PI548582	McCall	Glycine max	0	United States	Minnesota	45.18	-93.36	20.23	39.175
PI548593	Maple Arrow	Glycine max	0	Canada	Ontario	43.69	-79.4	21.32	40.04
PI548603	Perry	Glycine max	IV	United States	Indiana	39.78	-86.31	21.15	41.6
PI548604	Pershing	Glycine max	IV	United States	Missouri	38.67	-90.4	18.6	42.75
PI548619	Sparks	Glycine max	IV	United States	Kansas	39.11	-94.7	20.27	40.6
PI548631	Williams	Glycine max	III	United States	Illinois	39.76	-89.57	19.8	45.6
PI548633	Wye	Glycine max	IV	United States	Maryland	39.29	-76.7	19.2	41.3
PI548634	Zane	Glycine max	III	United States	Ohio	39.94	-83.09	21.6	40.55
PI548638	OAC Libra	Glycine max	0	Canada	Ontario	43.69	-79.4	21.45	40.95
PI548643	Maple Glen	Glycine max	0	Canada	Ontario	43.69	-79.4	20.4	42.6
PI548644	OAC Musca	Glycine max	0	Canada	Ontario	43.69	-79.4	21.1	39.85
PI548656	Lee	Glycine max	VI	United States	Mississippi	32.24	-90.21	20.25	45.15
PI548657	Jackson	Glycine max	VII	United States	North Carolina	35.22	-80.76	19.8	41.9
PI548667	Essex	Glycine max	V	United States	Virginia	37.52	-77.54	19.97	43.833
PI548978	Gail	Glycine max	VI	United States	Texas	29.73	-95.58	18.9	47.2
PI549017	ZYD 3938	Glycine max	IV	China	Ningxia	37.33	106.04	19.5	42.3
PI549018	ZYD 3939	Glycine max	V	China	Ningxia	37.33	106.04	10.1	48.7
PI549021A	Na hei dou	Glycine max	III	China	Liaoning	41.2	122.34	16.3	47.35
PI549028	Feng da li	Glycine max	V	China	Liaoning	41.2	122.34	19.6	44.5
PI549031		Glycine max	III	China	Beijing	40.19	116.2	14	41.1
PI549032	ZYD 2632	Glycine soja	III	China	Liaoning	40.55	124.07	10.4	46.4
PI549040	ZYD 2704	Glycine max	IV	China	Liaoning	40.55	124.07	20.7	42.1
PI549041A	ZYD 2709	Glycine max	III	China	Liaoning	41.2	122.34	11.8	45.5
PI549046	ZYD 3728	Glycine soja	IV	China	Shaanxi	37.53	107.4	16.5	41.9
PI549047	Mrs. Wong's Picn	Glycine soja	III	China	Beijing	40.22	116.43	15.1	42.9
PI549048		Glycine soja	III	China	Beijing	40.19	116.2	16	44.9
PI553047	Gordon	Glycine max	VII	United States	Georgia	33.74	-84.42	20.1	41
PI556949	Ke Feng No.1	Glycine max	IV	China	Beijing	40.38	116.39	16.9	38
PI559932	Manokin	Glycine max	IV	United States	Maryland	39.29	-76.7	20.64	42.06
PI561271	Pei xian da quing	Glycine max	V	China	Zhejiang	30.27	120.08	18.5	45.1
PI561318A	Hui nan bai hua x	Glycine max	I	China	Beijing	40.38	116.39	18.37	44.1
PI561370	Fen dou 14	Glycine max	III	China	Shanxi	37.33	111.83	18.7	42.8
PI561371	Fen dou 15	Glycine max	IV	China	Shanxi	37.33	111.83	20.7	43.75
PI561387	Kosuzu	Glycine max	V	Japan	Tokyo	35.73	139.57	19.6	41.3
PI561389B	(Okura Natto)	Glycine max	0	Japan	Tokyo	35.73	139.57	13.7	49
PI562534	KA5	Glycine soja	Unknow	Korea, South	Kyonggi	37.23	126.93	9.8	53.9
PI562547	KC1	Glycine soja	V	Korea, South	Chungchong N	36.57	126.68	12.5	45.3
PI562551	KC26	Glycine soja	V	Korea, South	Chungchong N	36.57	126.68	11.1	48.7
PI562553	KD5	Glycine soja	V	Korea, South	Chungchong N	36.18	126.57	10	50.8
PI562557	KE10	Glycine soja	Unknow	Korea, South	Cholla Puk	35.82	127.12	11.1	48.7
PI562558	KE12	Glycine soja	Unknow	Korea, South	Cholla Puk	35.82	127.12	11.4	49
PI562559	KE16	Glycine soja	Unknow	Korea, South	Cholla Puk	35.82	127.12	10.5	49.8
PI562565	KF13	Glycine soja	iV	Korea, South	Cholla Puk	35.53	127.33	12.7	47.3
PI562568	KF24	Glycine soja	Unknow	Korea, South	Cholla Puk	35.53	127.33	9.6	51.5
PI567071A	MARIF 2669	Glycine max	Unknow	Indonesia	Indonesia	-3.25	121.77	17.2	44.4

PI567164	He Long You Tai	Glycine max	0	China	Jilin	44.23	126	20.1	42.6
PI567171	Hei he No. 1	Glycine max	0	China	Heilongjiang	46.86	126.85	22.1	41.85
PI567189A	Ekhabac	Glycine max	IV	Vietnam	Vietnam	21	105	17	47.4
PI567225	Kisinevskaja 90	Glycine max	0	Moldova		47	29	19.7	43.7
PI567226	Har'kovskaja Zern	Glycine max	0	Russian Federation		52.98	127.36	15.6	44.7
PI567230	WJK-PRC-23	Glycine max	V	China	Shaanxi	37.53	107.4	11.9	46.6
PI567231	WJK-PRC-46	Glycine max	VIII	China	Sichuan	30.59	104.1	14	47.2
PI567258	He pi dou	Glycine max	II	China	Jiangxi	28.68	115.96	15	48.6
PI567262A	Similar to: Gu tian	Glycine max	II	China	Fujian	26.06	119.39	15.6	47.4
PI567298	Chan yao dou	Glycine max	V	China	Gansu	36.03	103.84	15.5	45.7
PI567305	Hei dou zi	Glycine max	IV	China	Gansu	36.03	103.84	14.85	42.6
PI567307	Hei huang dou	Glycine max	IV	China	Gansu	36.03	103.84	15.55	41.35
PI567336B	(Lao hei dou)	Glycine max	IV	China	Gansu	36.03	103.84	13.6	43.5
PI567343	Ma huang dou	Glycine max	V	China	Gansu	36.03	103.84	11.9	48
PI567346	Niu mao huang do	Glycine max	V	China	Gansu	36.03	103.84	13.4	45.2
PI567352A	Yang yan qing dou	Glycine max	IV	China	Gansu	36.03	103.84	17.8	41.6
PI567353	Yang yan ren dou	Glycine max	IV	China	Gansu	36.03	103.84	17.5	41.85
PI567354	You huang dou	Glycine max	IV	China	Gansu	36.03	103.84	18.2	42.85
PI567357	Du jia qiao huang	Glycine max	III	China	Ningxia	37.33	106.04	18.5	48.5
PI567361	Lu fang huang do	Glycine max	III	China	Ningxia	37.33	106.04	15.9	50.7
PI567364	Ping luo huang da	Glycine max	III	China	Ningxia	37.33	106.04	19.4	46.4
PI567368	Xi he huang dou	Glycine max	IV	China	Ningxia	37.33	106.04	15.8	50.45
PI567383	Da ke huang dou	Glycine max	V	China	Shaanxi	37.53	107.4	22.55	42.45
PI567395	Lai wa dou	Glycine max	IV	China	Shaanxi	37.53	107.4	15.1	46.8
PI567407	Xiao dou	Glycine max	V	China	Shaanxi	37.53	107.4	13.6	44
PI567408	Xiao jin huang	Glycine max	V	China	Shaanxi	37.53	107.4	17.6	45.5
PI567410B	(Yang huang dou)	Glycine max	VII	China	Shaanxi	37.53	107.4	16.7	46.9
PI567414	Zhu Ye Qing	Glycine max	V	China	Shaanxi	37.53	107.4	20.3	44.8
PI567415A	Bai da huang dou	Glycine max	IV	China	Shanxi	37.33	111.83	19.2	43.4
PI567416	Bai dou	Glycine max	IV	China	Shanxi	37.33	111.83	16.7	47.6
PI567418A	Bai hei dou	Glycine max	II	China	Shanxi	37.33	111.83	19.6	48.3
PI567426	Bai huang dou	Glycine max	IV	China	Shanxi	37.33	111.83	16.55	47.85
PI567428	Bai ji yao	Glycine max	IV	China	Shanxi	37.33	111.83	19.6	42.1
PI567435B	(Hei hei dou)	Glycine max	III	China	Shanxi	37.33	111.83	19	45.3
PI567439	Hong jia huang do	Glycine max	V	China	Shanxi	37.33	111.83	16.8	46.2
PI567488A	Di liu huang dou N	Glycine max	IV	China	Hebei	39.94	119.59	17.5	47.2
PI567489A	Er da li huang dou	Glycine max	IV	China	Hebei	39.94	119.59	16.9	46.8
PI567503	Niu mao huang	Glycine max	IV	China	Hebei	39.94	119.59	18.5	44
PI567505	Tu Er Yan	Glycine max	III	China	Hebei	39.94	119.59	19.7	44.9
PI567516C	(Ba yue zha)	Glycine max	IV	China	Shandong	36.6	118.97	17.8	42.6
PI567525	Cao qing huang do	Glycine max	II	China	Shandong	36.6	118.97	18.6	46.7
PI567532	Dai ye xiao huang	Glycine max	IV	China	Shandong	36.6	118.97	18.9	45.95
PI567548	Hua li hu zi	Glycine max	IV	China	Shandong	36.6	118.97	17.4	45.15
PI567576	Ping ding huang	Glycine max	III	China	Shandong	36.6	118.97	16.7	50.2
PI567604A	Xin huang dou	Glycine max	IV	China	Shandong	36.6	118.97	19.5	43.8
PI567611	Ba yue zha	Glycine max	IV	China	Henan	34.74	113.66	17.4	48.4
PI567651	Shang cai er cao p	Glycine max	IV	China	Henan	34.74	113.66	17	48.8

PI567675	Yu cheng xiao tie j	Glycine max	IV	China	Henan	34.74	113.66	18.4	44.6
PI567690	Fu yang (7)	Glycine max	III	China	Anhui	31.82	117.15	16.8	49.2
PI567698A	Fu yang (17)	Glycine max	IV	China	Anhui	31.82	117.15	15.4	49.2
PI567700	Fu yang (19)	Glycine max	III	China	Anhui	31.82	117.15	16.4	49
PI567719	Fu yang (43)	Glycine max	IV	China	Anhui	31.82	117.15	16.5	46.9
PI567720A	Fu yang (44)	Glycine max	III	China	Anhui	31.82	117.15	18.1	47.6
PI567726	Fu yang (50)	Glycine max	IV	China	Anhui	31.82	117.15	20.7	42.1
PI567730	Fu yang (54)	Glycine max	IV	China	Anhui	31.82	117.15	15.6	50
PI567731	Fu yang (56)	Glycine max	III	China	Anhui	31.82	117.15	17.8	47.4
PI567746	Pei xian da bai jiao	Glycine max	IV	China	Jiangsu	32.06	118.85	18.1	46.3
PI567756	Pei Xian Si Li Cao	Glycine max	IV	China	Jiangsu	32.06	118.85	**15.9**	**47.9**
PI567780B	(Tong shan zheng	Glycine max	IV	China	Jiangsu	32.06	118.85	17.6	46
PI567782	OAC Dorado	Glycine max	I	Canada	Ontario	43.69	-79.4	22.07	41
PI567788	Bienville	Glycine max	VIII	United States	Louisiana	30.4	-91.24	19	43.8
PI574477	Fen dou 31	Glycine max	IV	China	Shanxi	37.33	111.83	21	43.9
PI574482	Jin Dou No.2	Glycine max	III	China	Shanxi	37.33	111.83	19.1	45
PI574486	Jin dou 13	Glycine max	III	China	Shanxi	37.33	111.83	19.3	46.9
PI578309	I-64	Glycine max	VI	Nepal	Jumia	28.15	84.12	16.95	43.4
PI578341	L 521	Glycine soja	Unknown	Russian Feder	Khabarovsk	48.5	135.13	11.7	43.8
PI578357	KZ-6352/91	Glycine soja	Unknown	Russian Feder	Amur	52.98	127.36	15.6	43.5
PI578360	Guan Nan Chun H	Glycine max	II	China	Jiangsu	32.06	118.85	17.4	44.8
PI578375B	(Aan tu dang di he	Glycine max	I	China		40.38	116.39	16	50.7
PI578412	Gong jiao 6308-1	Glycine max	II	China		40.38	116.39	18.7	41.5
PI578494A	Jin Dou No.1	Glycine max	IV	China	Shanxi	37.33	111.83	18.5	41.7
PI578495	Jin dou No. 4	Glycine max	IV	China	Beijing	40.38	116.39	18.3	42.3
PI578503	Tie jia si li huang	Glycine max	I	China		40.38	116.39	17.7	39.8
PI578504	Xiang dou No. 3	Glycine max	II	China		40.38	116.39	17.35	46.1
PI587584	Yi zheng da li hua	Glycine max	VI	China	Jiangsu	32.06	118.85	18.35	44.15
PI587588A	Tai xing niu mao h	Glycine max	IV	China	Jiangsu	32.06	118.85	16.4	47.3
PI587588B	(Tai xing niu mao	Glycine max	V	China	Jiangsu	32.06	118.85	17.8	45.8
PI587666	Er dao zao	Glycine max	VI	China	Anhui	31.82	117.15	18	45.85
PI587712B	(E dou No. 1)	Glycine max	V	China	Hubei	30.59	114.16	18.85	45.65
PI587752	Xian ning dong hu	Glycine max	V	China	Hubei	30.59	114.16	19.4	44.6
PI587804	Jing 789	Glycine max	IV	China	Hubei	30.59	114.16	15.65	44.35
PI587811A	ZDD005777	Glycine max	VIII	China	Hubei	30.59	114.16	15	48.6
PI587848	Wu chang hei don	Glycine max	V	China	Hubei	30.59	114.16	19.15	36.7
PI588053A	Xiao li huang	Glycine max	V	China	Guangdong	23.12	113.25	18.25	44.4
PI58955	Common Yellow V	Glycine max	IV	China	Shandong	36.71	119.1	20.6	41.1
PI591431	OT94-49	Glycine max	0	Canada	Ontario	43.69	-79.4	19.9	43.9
PI591432	OT94-51	Glycine max	0	Canada	Ontario	51.37	-84.74	20.5	39.9
PI591433	OT94-37	Glycine max	0	Canada	Ontario	43.69	-79.4	19.5	43.5
PI591435	OT94-41	Glycine max	I	Canada	Ontario	43.69	-79.4	19.2	43.3
PI591495	L93-2740	Glycine max	IV	United States	Illinois	39.76	-89.57	20.6	42.1
PI591541	L74-102	Glycine max	II	United States	Illinois	39.76	-89.57	21.4	42
PI592523	Glacier	Glycine max	0	United States	Minnesota	45.18	-93.36	19	40.9
PI592937	Jin dou 14	Glycine max	IV	China		40.38	116.39	16.9	42.5
PI592940	Jin dou 17	Glycine max	IV	China		40.38	116.39	17.6	42.7

PI592952	Zheng 77249	Glycine max	III	China		40.38	116.39	19.7	42.05
PI592954	Nin zhen No. 1	Glycine max	II	China		40.38	116.39	19.1	40.3
PI592960	Dong nong 38	Glycine max	I	China	Heilongjiang	46.86	126.85	20.9	38.8
PI593258	Macon	Glycine max	III	United States	Illinois	39.76	-89.57	19.4	40.2
PI593953	Sui nong No. 10	Glycine max	I	China		40.38	116.39	19.35	39.7
PI593983	Hidaka-6	Glycine soja	III	Japan	Hokkaido	42.87	142.44	12	45.6
PI594012	Heuksatangdu	Glycine max	V	Korea, South	Korea	35.87	128.68	18.4	44.9
PI594170B	(Geden shirazu)	Glycine max	I	Japan	Akita	39.57	140.42	18.9	39.05
PI594301	Toyomusume	Glycine max	I	Japan	Japan	35.94	139.4	18.55	41.45
PI594307	Tsurusengoku	Glycine max	VIII	Japan	Tokyo	35.73	139.57	13.8	49.8
PI594393	Shui Niu Pi	Glycine max	IV	China	Anhui	31.82	117.15	17.9	42.3
PI594451	Liu yue bao	Glycine max	III	China	Sichuan	30.59	104.1	15.2	46
PI594456A	Xiao jin huang	Glycine max	III	China	Sichuan	30.59	104.1	16.5	46
PI594512A	Bian zi jiang se do	Glycine max	VII	China	Sichuan	30.59	104.1	17.05	47.45
PI594579	Zhong he tian che	Glycine max	V	China	Hunan	28.24	112.81	18.4	45.65
PI594599	Chang de chun he	Glycine max	IV	China	Hunan	28.24	112.81	14.7	44.7
PI594615	Liu yue zao	Glycine max	IV	China	Guizhou	26.65	106.69	16.55	47.05
PI594629	Xiao hua lian	Glycine max	VI	China	Guizhou	26.65	106.69	14.5	49.5
PI594788	Da zao dou	Glycine max	Unknown	China	Yunnan	24.9	102.96	19.9	46.3
PI594880	Song zi dou	Glycine max	V	China	Yunnan	24.9	102.96	15.7	46.5
PI594922	Graham	Glycine max	V	United States	North Carolina	35.22	-80.76	20.93	40.267
PI597464	Zhe chun No. 3	Glycine max	II	China	Zhejiang	30.27	120.08	16.9	44.9
PI597476	Deogyukong	Glycine max	V	Korea, South	Korea	35.87	128.68	18.8	49
PI597478B	(Paldalkong)	Glycine max	III	Korea, South	Korea	35.87	128.68	16.9	42.6
PI598124	Maverick	Glycine max	III	United States	Missouri	38.67	-90.4	18.7	41.7
PI598358	TN 5-95	Glycine max	V	United States	Tennessee	36.18	-86.81	18.8	42.4
PI59845	Sohgetsu	Glycine max	VI	Japan	Akita	39.45	140.48	18.6	42.45
PI602502B	(Xiong yue xiao hu	Glycine max	IV	China		40.38	116.39	16.5	46.3
PI602991	Niu jiao qi da hei d	Glycine max	IV	China	Shandong	36.6	118.97	15.6	44.3
PI602993	Pi xian ruan tiao z	Glycine max	IV	China	Jiangsu	32.06	118.85	15.3	45.7
PI602994	Pu Dong Da Huan	Glycine max	VII	China	Shanghai	31.15	121.8	16.6	45.1
PI603154	GL 2622 /96	Glycine max	V	Korea, North		39.04	125.75	19.6	45.6
PI603162	GL 2631 /96	Glycine max	IV	Korea, North		39.04	125.75	14.5	50.2
PI603170	GL 2683 /96	Glycine max	IV	Korea, North		39.04	125.75	12.8	49.6
PI603175	GL 2688 /96	Glycine max	IV	Korea, North		39.04	125.75	13.6	48.2
PI603176A		Glycine max	IV	Korea, North		39.04	125.75	14.6	51.2
PI603290	Zao shu 18	Glycine max	I	China		40.38	116.39	19.6	40.533
PI603294	Jin Yuan No.2	Glycine max	0	China	Heilongjiang	46.86	126.85	18	43.4
PI603318	Xiao zhu yao	Glycine max	I	China	Heilongjiang	46.86	126.85	19.1	43.6
PI603336	Qing pi si li huang	Glycine max	II	China	Heilongjiang	46.86	126.85	15.9	46.9
PI603345	ZDD00403	Glycine max	II	China		40.38	116.39	19	41
PI603357	Du Lu Dou	Glycine max	I	China	Jilin	44.23	126	18.85	40.3
PI603384	Ping ding xiang	Glycine max	III	China	Jilin	44.23	126	17.3	43.9
PI603389	Huang ke	Glycine max	II	China		40.38	116.39	18	43
PI603397	Hei qi huang da d	Glycine max	IV	China		40.38	116.39	15.1	46.6
PI603413	Yu shi dou	Glycine max	IV	China	Liaoning	41.2	122.34	17	48.8
PI603420	ZDD01501	Glycine max	II	China	Innermongolia	40.8	111.87	16.9	42.5

PI603424A	ZDD007871	Glycine max	0	China	China	40.38	116.39	18.3	41.9
PI603426G	(Ben di yuan huar	Glycine max	II	China		40.38	116.39	16.9	41.2
PI603442	Ke qi xiao hei dou	Glycine max	III	China		40.38	116.39	15	44.7
PI603458A	Shui dou	Glycine max	IV	China		40.38	116.39	15.8	43.5
PI603463	Dong jie No. 1	Glycine max	II	China		40.38	116.39	18.1	42.5
PI603469	Pa Man Qing	Glycine max	IV	China	Shandong	36.6	118.97	17.7	44.8
PI603488	ZDD19294	Glycine max	III	China		40.38	116.39	17.6	43.45
PI603492	Qi hei dou	Glycine max	IV	China		40.38	116.39	15.7	44.9
PI603494	Hai dou zi	Glycine max	IV	China		40.38	116.39	15.9	42.8
PI603495B	(Hong mi lan dou	Glycine max	V	China		40.38	116.39	15.3	46.2
PI603497	Hua dou	Glycine max	III	China		40.38	116.39	12.2	49.2
PI603516	Xiao ma yi dan	Glycine max	VI	China	Shaanxi	37.53	107.4	11	51.3
PI603526	Hei you dou	Glycine max	IV	China		40.38	116.39	16.3	42.8
PI603549	Mei dou	Glycine max	III	China		40.38	116.39	19.2	44.9
PI603555	Hua da hei dou	Glycine max	IV	China		40.38	116.39	17.5	45.8
PI603556	ZDD08563	Glycine max	III	China		40.38	116.39	15.6	45.1
PI603559	ZDD08590	Glycine max	IV	China		40.38	116.39	15.8	45.6
PI603675	Huai yin gua dou j	Glycine max	III	China	Jiangsu	32.06	118.85	17	46.9
PI603698J	(Dan yang shui ba	Glycine max	0	China		40.38	116.39	15.9	45.8
PI603722	Nan chong ba yue	Glycine max	VIII	China		40.38	116.39	12.1	53.4
PI603756	ZDD05996	Glycine max	II	China	Zhejiang	30.27	120.08	16.3	46.4
PI605869A	Sample 140	Glycine max	V	Vietnam	Lao Cai	21	103.5	12.3	49.3
PI612611	Browngilgun	Glycine max	III	Korea, North		39.04	125.75	16.9	44.6
PI612719	Harbin 91-6065	Glycine max	I	China	Heilongjiang	46.86	126.85	18.9	40.4
PI612730	Zhong huong No.	Glycine max	II	China		40.38	116.39	20	39.8
PI612733	Ji Qing No.1	Glycine max	II	China	Jilin	44.23	126	20.8	43.2
PI612754	ZY 645	Glycine max	I	China		40.38	116.39	13.95	47
PI62203	937	Glycine max	V	China	Hebei	39.94	119.59	20	42.6
PI68521-1	205	Glycine max	Unknow	China	Duitsinshan, M	46.2	126.07	21.3	42.3
PI68604-1	285	Glycine max	Unknow	China	Yaomyn, Manc	46.2	126.07	19.85	42.4
PI68732-1	204	Glycine max	Unknow	China	Heilongjiang	46.64	125.43	20.2	41.6
PI70080	6908	Glycine max	III	China	Jilin	43.71	128.24	19.6	42.8
PI70466-3	7336	Glycine max	Unknow	China	Jilin	41.94	126.43	19.9	43.9
PI71465	No. 33	Glycine max	V	China	Jiangsu	32.06	118.85	20.55	44.35
PI79691-4		Glycine max	III	China	Heilongjiang	45.75	126.65	18.5	42.6
PI80822	Shiheigai Shiroba	Glycine max	III	China	Liaoning	41.2	122.34	19.9	44
PI80837	Mejiro	Glycine max	IV	Japan	Akita	39.57	140.42	18.6	44.5
PI81041	Kuro Daizu	Glycine max	III	Japan	Hokkaido	43.07	141.35	19.9	42.8
PI81775	1486	Glycine max	I	Japan	Akita	39.45	140.48	19	41.5
PI81785	Chusei Hadaka	Glycine max	III	Japan	Hokkaido	43.07	141.3	17.2	43.3
PI83881	Orukon	Glycine max	IV	Korea, North	Kangwon	39.15	127.44	18.7	43.1
PI83942	Kuro churyu	Glycine max	V	Korea, South	Kyonggi	37.28	127.12	19.4	45.55
PI84631	S-56	Glycine max	III	Korea, South	Kyonggi	37.28	127.12	19.7	42.4
PI84637	S-62	Glycine max	II	Korea, South	Kyonggi	37.28	127.12	20.1	41.2
PI84656	S-81	Glycine max	III	Korea, South	Kyonggi	37.28	127.12	21.7	42.4
PI84946-2	(Kandokon)	Glycine max	Unknow	Korea, South	Pusan	35.15	129.05	20.4	42.9
PI84973	Takiya	Glycine max	III	Japan	Saitama	35.85	139.65	19.2	43.3

PI84987	Oni Hadaka	Glycine max	III	Japan	Saitama	35.85	139.65	19.1	41.4
PI84987A	(Oni Hadaka)	Glycine max	III	Japan	Saitama	35.85	139.65	18.7	43.1
PI86006	Kiio Shokuzu	Glycine max	III	Japan	Hokkaido	42.92	143.2	16.75	45.15
PI86024	Daidzuhinshu sat	Glycine max	III	Japan	Hokkaido	42.92	143.2	18.7	46.5
PI86904	Fukota	Glycine max	IV	Korea, South	Chungchong Pu	37	128.17	16.2	44.9
PI86972-2	(Pakute)	Glycine max	Unknow	Korea, South	Cholla Puk	35.95	126.96	18.9	40.5
PI87617	Miyongaikon	Glycine max	III	Korea, North	Hamgyong Puk	41.67	129.66	21	42.5
PI87620	Kuromeshoryu	Glycine max	IV	Korea, North	Hamgyong Puk	41.67	129.66	19.9	42.5
PI87631-1	Kindaizu	Glycine max	III	Japan	Saitama	36.13	139.38	18.1	41.5
PI88313	5702	Glycine max	II	China	Chirin, Manchu	49.59	117.38	21.6	37.3
PI88468	Iganzu	Glycine max	II	China	Liaoning	40.18	122.12	22	39.55
PI88479	Kungchuling Impr	Glycine max	II	China	Jilin	44.23	126	22.45	38.55
PI88788	5913	Glycine max	III	China	Liaoning	42.72	124.33	17.5	42.4
PI89005-5	5950	Glycine max	Unknow	China	Manchuria	46.2	126.07	21.15	42.6
PI89138	Zontanorukon	Glycine max	II	Korea, North	Hamgyong Puk	41.67	129.66	20.65	41.15
PI89772	7193	Glycine max	IV	China		40.38	116.39	17.5	41.7
PI89775	7221	Glycine max	VI	China	Near Fa Hua Ss	24.65	113.64	16.5	45.6
PI90479P	7413	Glycine max	IV	China	Nanchuangying	40.38	116.39	18.65	43.85
PI90763	7570	Glycine max	IV	China	Beijing	39.91	116.6	18.9	40.5
PI91100-3	6554	Glycine max	Unknow	China	Jilin	43.51	124.81	20	40.6
PI91159-4	6614	Glycine max	Unknow	China	Liaoning	42.72	124.33	20.1	43.2
PI91160	6615	Glycine max	III	China	Liaoning	42.72	124.33	19.6	42.3
PI92651	7846	Glycine max	IV	China	Jilin	43.51	124.81	19.7	39.9
PI94159-3	(Kiizaya)	Glycine max	Unknow	Japan	Kagoshima	31.33	130.93	18.7	42.6
