# Supplementary material for: Selection of GmSWEET39 for oil and protein improvement in soybean
Source: PLoS Genet. 2020 Nov 11;16(11):e1009114. doi: 10.1371/journal.pgen.1009114 (PMC7721174; doi:10.1371/journal.pgen.1009114)
Supplement: S2 Table — (PDF) [file pgen.1009114.s004.pdf]

S2 Table The most signficantly associated DNA variants for oil and protein

DNA Variants			Oil Content		Protein Conent	
**ID**	**Chr**	**Position**	**P value**	**MarkerR2**	**P value**	**MarkerR2**
DT15_3875621	15	3875621	1.81E-07	0.04367	9.72E-07	0.05751
DT15_3875101	15	3875101	2.99E-06	0.04158	9.16E-06	0.03758
DA15_3874703	15	3874703	3.12E-06	0.03355	1.21E-06	0.04487
