# Supplementary material for: Selection of GmSWEET39 for oil and protein improvement in soybean
Source: PLoS Genet. 2020 Nov 11;16(11):e1009114. doi: 10.1371/journal.pgen.1009114 (PMC7721174; doi:10.1371/journal.pgen.1009114)
Supplement: S3 Table — (PDF) [file pgen.1009114.s005.pdf]

**S3 Table Haplotypes of the accessions identified in this study**

| PI | CommonName | Species | MATGROUP | Country | Origin_State_Province | latitude | longitude | Haplotype |
|---|---|---|---|---|---|---|---|---|
| FC33243 | Anderson | G. max | IV | Unknown | Iowa | 41.56 | -93.5 | H1 |
| PI123440 | No. 2 | G. max | VI | Myanmar | | 19.71 | 95.37 | H1 |
| PI153262 | Roumanie | G. max | 0 | Belgium | Belgium | 40.38 | 116.39 | H1 |
| PI180501 | Strain No. 18 | G. max | 0 | Germany | | 52.51 | 13.41 | H1 |
| PI196166 | No. 2296 | G. max | V | Korea, South | Korea | 35.87 | 128.68 | H1 |
| PI200508 | Natsu Daizu | G. max | I | Japan | Shikoku | 33.74 | 133.66 | H1 |
| PI209332 | No. 4 | G. max | IV | Japan | Hokkaido | 43.46 | 142.61 | H1 |
| PI243541 | Shakujo | G. max | IV | Japan | Akita | 39.57 | 140.42 | H1 |
| PI248515 | White Hilum Iwata | G. max | IV | Japan | Northern part of H | 36.12 | 138.12 | H1 |
| PI253658B | No. 9 | G. max | I | China | China | 40.38 | 116.39 | H1 |
| PI317336 | Shinsei | G. max | 0 | Japan | Hokkaido | 43.46 | 142.61 | H1 |
| PI398881 | KLS 126-1 | G. max | III | Korea, South | Kyonggi | 37 | 127 | H1 |
| PI399043 | KLS 903 | G. max | III | Korea, South | Cheju | 33.4 | 126.55 | H1 |
| PI404188A | Huaj an si er dian | G. max | II | China | N/A | 40.38 | 116.39 | H1 |
| PI407716 | Jin nung No. 3 | G. max | I | China | Jilin | 44.23 | 126 | H1 |
| PI407849 | KAS 510-1 | G. max | III | Korea, South | Cholla Puk | 35.95 | 126.96 | H1 |
| PI408105A | KAS 633-19 | G. max | IV | Korea, South | Kyongsang Puk | 36.08 | 129.36 | H1 |
| PI416937 | Houjaku Kuwazu | G. max | VI | Japan | Kanto and Tosan re | 36 | 139 | H1 |
| PI416971 | Kaifuu gyuumou ou | G. max | IV | Japan | Kinki | 34.7 | 135.5 | H1 |
| PI437169B | (VNIISC-4) | G. max | II | Russian Federation | Krasnodar | 45.1 | 39.27 | H1 |
| PI437655 | Er-huan-jan | G. max | III | China | | 40.38 | 116.39 | H1 |
| PI437725 | Te-zu-gan | G. max | IV | China | | 40.38 | 116.39 | H1 |
| PI437863A | DV-2841 | G. max | II | China | Northeast | 40.38 | 116.39 | H1 |
| PI438471 | Fiskeby III | G. max | 0 | Sweden | Ostergotland | 58.4 | 15.66 | H1 |
| PI467343 | Yan-nong No. 2 | G. max | I | China | Jilin | 44.23 | 126 | H1 |
| PI471938 | 197 | G. max | V | Nepal | Jumia | 28.13 | 84.14 | H1 |
| PI475783B | (Tsing 2) | G. max | III | China | Shanxi | 37.33 | 111.83 | H1 |
| PI483252 | Doko | G. max | IX | Brazil | | -14 | -50 | H1 |
| PI495017C | (Beijing da qing dor | G. max | IV | China | Beijing | 40.38 | 116.39 | H1 |
| PI507354 | Tokei 421 | G. max | I | Japan | Hokkaido | 43.46 | 142.61 | H1 |
| PI507355 | Tokei 423 | G. max | I | Japan | Hokkaido | 43.46 | 142.61 | H1 |
| PI507681B | (Jangiabad) | G. max | II | Uzbekistan | N/A | 41.31 | 69.25 | H1 |
| PI508083 | Dassel | G. max | 0 | United States | Minnesota | 45.18 | -93.36 | H1 |
| PI508266 | Young | G. max | VI | United States | North Carolina | 35.22 | -80.76 | H1 |
| PI513382 | Glenwood | G. max | 0 | United States | Minnesota | 45.18 | -93.36 | H1 |
| PI515961 | Pennyrile | G. max | IV | United States | Kentucky | 38.2 | -85.76 | H1 |
| PI518703 | He feng 25 | G. max | I | China | Heilongjiang | 46.83 | 130.35 | H1 |
| PI518711 | Jilin 21 | G. max | II | China | Jilin | 43.51 | 124.81 | H1 |
| PI518751 | NS-20 | G. max | II | Former Serbia and | N/A | 44.02 | 21.15 | H1 |
| PI533655 | Burlison | G. max | II | United States | Illinois | 39.76 | -89.57 | H1 |
| PI540556 | Jack | G. max | II | United States | Illinois | 39.76 | -89.57 | H1 |
| PI542044 | Kunitz | G. max | III | United States | Illinois | 39.76 | -89.57 | H1 |

| | | | | | | | | |
|---|---|---|---|---|---|---|---|---|
| PI542403 | Dawson | G. max | 0 | United States | Minnesota | 45.18 | -93.36 | H1 |
| PI547460 | L64-1083 | G. max | IV | United States | Illinois | 39.76 | -89.57 | H1 |
| PI547690 | L63-1212 | G. max | II | United States | Illinois | 39.76 | -89.57 | H1 |
| PI547716 | L62-667 | G. max | II | United States | Illinois | 39.76 | -89.57 | H1 |
| PI548298 | A.K. (Harrow) | G. max | III | China | China | 40.38 | 116.39 | H1 |
| PI548311 | Capital | G. max | 0 | Canada | Ontario | 43.69 | -79.4 | H1 |
| PI548316 | Cloud | G. max | III | China | Zhejiang | 30.27 | 120.08 | H1 |
| PI548342 | Higan | G. max | IV | Japan | Tokyo | 35.73 | 139.57 | H1 |
| PI548348 | Illini | G. max | III | China | Northeast | 40.38 | 116.39 | H1 |
| PI548349 | Ilsoy | G. max | III | Korea, North | Pyongyang | 39.04 | 125.75 | H1 |
| PI548356 | Kanro | G. max | II | Korea, North | Pyongyang | 39.04 | 125.75 | H1 |
| PI548360 | Korean | G. max | II | Korea, North | | 39.04 | 125.75 | H1 |
| PI548379 | Mandarin (Ottawa) | G. max | 0 | China | Heilongjiang | 46.86 | 126.85 | H1 |
| PI548382 | Manitoba Brown | G. max | 0 | China | Liaoning | 41.2 | 122.34 | H1 |
| PI548391 | Mukden | G. max | II | China | Liaoning | 41.2 | 122.34 | H1 |
| PI548406 | Richland | G. max | II | China | Jilin | 44.23 | 126 | H1 |
| PI548417 | Soysota | G. max | I | Italy | Naples | 40.85 | 14.26 | H1 |
| PI548456 | Haberlandt | G. max | VI | Korea, North | Pyongyang | 39.04 | 125.75 | H1 |
| PI548477 | Ogden | G. max | VI | United States | Tennessee | 36.18 | -86.81 | H1 |
| PI548488 | S-100 | G. max | V | China | Heilongjiang | 46.86 | 126.85 | H1 |
| PI548510 | Beeson | G. max | II | USA | Indiana | 39.78 | -86.31 | H1 |
| PI548511 | Beeson 80 | G. max | II | United States | Indiana | 39.78 | -86.31 | H1 |
| PI548512 | Century | G. max | II | United States | Indiana | 39.78 | -86.31 | H1 |
| PI548520 | Preston | G. max | II | United States | Iowa | 41.56 | -93.5 | H1 |
| PI548533 | Clark | G. max | IV | United States | Illinois | 39.76 | -89.57 | H1 |
| PI548540 | Corsoy | G. max | II | United States | Iowa | 41.56 | -93.5 | H1 |
| PI548565 | Gnome | G. max | II | United States | Ohio | 39.94 | -83.09 | H1 |
| PI548573 | Harosoy | G. max | II | Canada | Ontario | 43.69 | -79.4 | H1 |
| PI548582 | McCall | G. max | 0 | United States | Minnesota | 45.18 | -93.36 | H1 |
| PI548593 | Maple Arrow | G. max | 0 | Canada | Ontario | 43.69 | -79.4 | H1 |
| PI548603 | Perry | G. max | IV | United States | Indiana | 39.78 | -86.31 | H1 |
| PI548604 | Pershing | G. max | IV | United States | Missouri | 38.67 | -90.4 | H1 |
| PI548631 | Williams | G. max | III | United States | Illinois | 39.76 | -89.57 | H1 |
| PI548634 | Zane | G. max | III | United States | Ohio | 39.94 | -83.09 | H1 |
| PI548643 | Maple Glen | G. max | 0 | Canada | Ontario | 43.69 | -79.4 | H1 |
| PI548656 | Lee | G. max | VI | United States | Mississippi | 32.24 | -90.21 | H1 |
| PI548657 | Jackson | G. max | VII | United States | North Carolina | 35.22 | -80.76 | H1 |
| PI548667 | Essex | G. max | V | United States | Virginia | 37.52 | -77.54 | H1 |
| PI553047 | Gordon | G. max | VII | United States | Georgia | 33.74 | -84.42 | H1 |
| PI561271 | Pei xian da quing d | G. max | V | China | Zhejiang | 30.27 | 120.08 | H1 |
| PI561370 | Fen dou 14 | G. max | III | China | Shanxi | 37.33 | 111.83 | H1 |
| PI567071A | MARIF 2669 | G. max | Unknow | Indonesia | Indonesia | -3.25 | 121.77 | H1 |
| PI567298 | Chan yao dou | G. max | V | China | Gansu | 36.03 | 103.84 | H1 |
| PI567354 | You huang dou | G. max | IV | China | Gansu | 36.03 | 103.84 | H1 |
| PI567357 | Du jia qiao huang d | G. max | III | China | Ningxia | 37.33 | 106.04 | H1 |
| PI567383 | Da ke huang dou | G. max | V | China | Shaanxi | 37.53 | 107.4 | H1 |
| PI567395 | Lai wa dou | G. max | IV | China | Shaanxi | 37.53 | 107.4 | H1 |

| | | | | | | | | |
|---|---|---|---|---|---|---|---|---|
| PI567525 | Cao qing huang dou | G. max | II | China | Shandong | 36.6 | 118.97 | H1 |
| PI567690 | Fu yang (7) | G. max | III | China | Anhui | 31.82 | 117.15 | H1 |
| PI567719 | Fu yang (43) | G. max | IV | China | Anhui | 31.82 | 117.15 | H1 |
| PI567731 | Fu yang (56) | G. max | III | China | Anhui | 31.82 | 117.15 | H1 |
| PI567782 | OAC Dorado | G. max | I | Canada | Ontario | 43.69 | -79.4 | H1 |
| PI574486 | Jin dou 13 | G. max | III | China | Shanxi | 37.33 | 111.83 | H1 |
| PI587666 | Er dao zao | G. max | VI | China | Anhui | 31.82 | 117.15 | H1 |
| PI587752 | Xian ning dong hua | G. max | V | China | Hubei | 30.59 | 114.16 | H1 |
| PI588053A | Xiao li huang | G. max | V | China | Guangdong | 23.12 | 113.25 | H1 |
| PI591431 | OT94-49 | G. max | 0 | Canada | Ontario | 43.69 | -79.4 | H1 |
| PI591435 | OT94-41 | G. max | I | Canada | Ontario | 43.69 | -79.4 | H1 |
| PI591495 | L93-2740 | G. max | IV | United States | Illinois | 39.76 | -89.57 | H1 |
| PI591541 | L74-102 | G. max | II | United States | Illinois | 39.76 | -89.57 | H1 |
| PI594301 | Toyomusume | G. max | I | Japan | Japan | 35.94 | 139.4 | H1 |
| PI594512A | Bian zi jiang se dou | G. max | VII | China | Sichuan | 30.59 | 104.1 | H1 |
| PI594579 | Zhong he tian chen | G. max | V | China | Hunan | 28.24 | 112.81 | H1 |
| PI594599 | Chang de chun hei | G. max | IV | China | Hunan | 28.24 | 112.81 | H1 |
| PI598124 | Maverick | G. max | III | United States | Missouri | 38.67 | -90.4 | H1 |
| PI602991 | Niu jiao qi da hei do | G. max | IV | China | Shandong | 36.6 | 118.97 | H1 |
| PI603154 | GL 2622 /96 | G. max | V | Korea, North | | 39.04 | 125.75 | H1 |
| PI603318 | Xiao zhu yao | G. max | I | China | Heilongjiang | 46.86 | 126.85 | H1 |
| PI603336 | Qing pi si li huang | G. max | II | China | Heilongjiang | 46.86 | 126.85 | H1 |
| PI603357 | Du Lu Dou | G. max | I | China | Jilin | 44.23 | 126 | H1 |
| PI603384 | Ping ding xiang | G. max | III | China | Jilin | 44.23 | 126 | H1 |
| PI603675 | Huai yin gua dou jia | G. max | III | China | Jiangsu | 32.06 | 118.85 | H1 |
| PI612719 | Harbin 91-6065 | G. max | I | China | Heilongjiang | 46.86 | 126.85 | H1 |
| PI80822 | Shiheigai Shirobana | G. max | III | China | Liaoning | 41.2 | 122.34 | H1 |
| PI80837 | Mejiro | G. max | IV | Japan | Akita | 39.57 | 140.42 | H1 |
| PI84631 | S-56 | G. max | III | Korea, South | Kyonggi | 37.28 | 127.12 | H1 |
| PI84987 | Oni Hadaka | G. max | III | Japan | Saitama | 35.85 | 139.65 | H1 |
| PI87617 | Miyongaikon | G. max | III | Korea, North | Hamgyong Puk | 41.67 | 129.66 | H1 |
| PI88479 | Kungchuling Improv | G. max | II | China | Jilin | 44.23 | 126 | H1 |
| PI88788 | 5913 | G. max | III | China | Liaoning | 42.72 | 124.33 | H1 |
| PI89138 | Zontanorukon | G. max | II | Korea, North | Hamgyong Puk | 41.67 | 129.66 | H1 |
| PI90763 | 7570 | G. max | IV | China | Beijing | 39.91 | 116.6 | H1 |
| PI548364 | Macoupin | G. max | IV | Japan | Tokyo | 35.73 | 139.57 | H1 |
| PI548400 | Patoka | G. max | IV | China | Heilongjiang | 46.86 | 126.85 | H1 |
| PI548619 | Sparks | G. max | IV | United States | Kansas | 39.11 | -94.7 | H1 |
| PI559932 | Manokin | G. max | IV | United States | Maryland | 39.29 | -76.7 | H1 |
| FC29333 | Laredo | G. max | III | | | | | H1 |
| FC31697 | | G. max | IV | Costa Rica | | 9.91 | -84.2 | H1 |
| PI54591 | No. 31 | G. max | III | China | Liaoning | 41.2 | 122.34 | H1 |
| PI54615-1 | No. 55 | G. max | Unknow | China | Heilongjiang | 45.75 | 126.65 | H1 |
| PI58955 | Common Yellow Va | G. max | IV | China | Shandong | 36.71 | 119.1 | H1 |
| PI62203 | 937 | G. max | V | China | Hebei | 39.94 | 119.59 | H1 |
| PI68732-1 | 204 | G. max | Unknow | China | Heilongjiang | 46.64 | 125.43 | H1 |
| PI70080 | 6908 | G. max | III | China | Jilin | 43.71 | 128.24 | H1 |

| | | | | | | | | |
|---|---|---|---|---|---|---|---|---|
| PI70466-3 | 7336 | G. max | Unknov | China | Jilin | 41.94 | 126.43 | H1 |
| PI71465 | No. 33 | G. max | V | China | Jiangsu | 32.06 | 118.85 | H1 |
| PI81041 | Kuro Daizu | G. max | III | Japan | Hokkaido | 43.07 | 141.35 | H1 |
| PI83881 | Orukon | G. max | IV | Korea, North | Kangwon | 39.15 | 127.44 | H1 |
| PI83942 | Kuro churyu | G. max | V | Korea, South | Kyonggi | 37.28 | 127.12 | H1 |
| PI84637 | S-62 | G. max | II | Korea, South | Kyonggi | 37.28 | 127.12 | H1 |
| PI84656 | S-81 | G. max | III | Korea, South | Kyonggi | 37.28 | 127.12 | H1 |
| PI84946-2 | (Kandokon) | G. max | Unknov | Korea, South | Pusan | 35.15 | 129.05 | H1 |
| PI84973 | Takiya | G. max | III | Japan | Saitama | 35.85 | 139.65 | H1 |
| PI86904 | Fukota | G. max | IV | Korea, South | Chungchong Puk | 37 | 128.17 | H1 |
| PI86972-2 | (Pakute) | G. max | Unknov | Korea, South | Cholla Puk | 35.95 | 126.96 | H1 |
| PI87620 | Kuromeshoryu | G. max | IV | Korea, North | Hamgyong Puk | 41.67 | 129.66 | H1 |
| PI88313 | 5702 | G. max | II | China | Chirin, Manchuria | 49.59 | 117.38 | H1 |
| PI88468 | Iganzu | G. max | II | China | Liaoning | 40.18 | 122.12 | H1 |
| PI90479P | 7413 | G. max | IV | China | Nanchuangying | 40.38 | 116.39 | H1 |
| PI91100-3 | 6554 | G. max | Unknov | China | Jilin | 43.51 | 124.81 | H1 |
| PI91159-4 | 6614 | G. max | Unknov | China | Liaoning | 42.72 | 124.33 | H1 |
| PI91160 | 6615 | G. max | III | China | Liaoning | 42.72 | 124.33 | H1 |
| PI92651 | 7846 | G. max | IV | China | Jilin | 43.51 | 124.81 | H1 |
| PI94159-3 | (Kiizaya) | G. max | Unknov | Japan | Kagoshima | 31.33 | 130.93 | H1 |
| PI153231 | B-63 | G. max | III | | | | | H1 |
| PI154189 | No. 57 | G. max | 0 | Netherlands | | 52.32 | 4.89 | H1 |
| PI159925 | Glycine H | G. max | VIII | Peru | Lima | -12.05 | -77.04 | H1 |
| PI165675 | Nanking 332 | G. max | VII | China | Jiangsu | 32.06 | 118.85 | H1 |
| PI179935 | Bhart | G. max | VII | India | Himachal Pradesh | 31.88 | 77.39 | H1 |
| PI189873 | Miko Saumon | G. max | 0 | France | | 48.83 | 2.22 | H1 |
| PI209333 | No. 3 | G. max | VI | Japan | Hokkaido | 43.46 | 142.61 | H1 |
| PI240664 | Bilomi No. 3 | G. max | X | Philippines | Luzon | 15 | 121.27 | H1 |
| PI253661B | No. 12 | G. max | III | China | | 40.38 | 116.39 | H1 |
| PI266806C | No. 4 | G. max | II | China | Hebei | 39.94 | 119.59 | H1 |
| PI291294 | | G. max | I | China | Heilongjiang | 46.86 | 126.85 | H1 |
| PI291310C | | G. max | II | China | Heilongjiang | 45.75 | 126.65 | H1 |
| PI297505 | Czi ti No. 5 | G. max | I | China | | 40.38 | 116.39 | H1 |
| PI297520 | Iregi Universal | G. max | 0 | Hungary | | 47.49 | 19.09 | H1 |
| PI324924 | Rhosa | G. max | V | South Africa | | -29.3 | 26.12 | H1 |
| PI361070 | Faur | G. max | 0 | Romania | | 44.42 | 26.11 | H1 |
| PI361087 | Medias 23 | G. max | I | Romania | | 44.42 | 26.11 | H1 |
| PI372403B | (Caloria) | G. max | 0 | Austria | | 48.2 | 16.38 | H1 |
| PI372418 | Novosadska Br. 4 | G. max | I | Serbia | | 44.02 | 21.15 | H1 |
| PI378680E | (VNIIMK 9186) | G. max | I | Russian Federation | | 52.98 | 127.36 | H1 |
| PI379618 | TC 1 | G. max | V | Taiwan | Taiwan | 24.73 | 121.08 | H1 |
| PI391583 | Jilin No. 10 | G. max | II | China | Jilin | 44.23 | 126 | H1 |
| PI398965 | KLS 628-1 | G. max | IV | Korea, South | Cholla Nam | 34.5 | 126.38 | H1 |
| PI404187 | Suj nii hun mao ju | G. max | II | China | | 40.38 | 116.39 | H1 |
| PI407701 | Hei long No. 3 | G. max | I | China | | 40.38 | 116.39 | H1 |
| PI407742 | 16 | G. max | V | China | Shaanxi | 34.26 | 108.93 | H1 |
| PI416751 | A-B(D) | G. max | I | Japan | Tohoku region | 39 | 141 | H1 |

| | | | | | | | | |
|---|---|---|---|---|---|---|---|---|
| PI416838 | Choutan | G. max | V | Japan | Tohoku region | 39 | 141 | H1 |
| PI417215 | Ooita Aki Daizu 2 | G. max | VIII | Japan | Kyushu and Okinav | 26.54 | 128.02 | H1 |
| PI417242 | Pekin dai seitou | G. max | II | China | | 40.38 | 116.39 | H1 |
| PI417345B | (Shou outou) | G. max | IV | China | | 40.38 | 116.39 | H1 |
| PI417479 | Yougetsu | G. max | IV | Japan | Tohoku region | 39 | 141 | H1 |
| PI417500 | Escura A | G. max | VIII | Brazil | | -14 | -46.6 | H1 |
| PI424195A | ISZ-3 | G. max | 0 | Hungary | | 47.49 | 19.09 | H1 |
| PI430595 | 58-161 | G. max | IV | China | | 40.38 | 116.39 | H1 |
| PI436684 | Tie-feng 8 | G. max | III | China | Liaoning | 41.2 | 122.34 | H1 |
| PI437112A | VIR 249 | G. max | II | Russian Federatior | Jewish Region, Far | 48.6 | 132.3 | H1 |
| PI437127A | Imeretinscaja | G. max | IV | Georgia | | 42.31 | 43.37 | H1 |
| PI437240 | CSchi 1069 | G. max | 0 | Moldova | | 47 | 29 | H1 |
| PI437376A | Ussurijscaja 308 | G. max | I | Russian Federatior | Primorye | 45 | 135 | H1 |
| PI437485 | VIR 1048 | G. max | II | Russian Federatior | Primorye | 45 | 135 | H1 |
| PI437500A | VIR 3810 | G. max | I | Russian Federatior | Primorye | 45 | 135 | H1 |
| PI437685D | (Phun-zhun) | G. max | III | China | | 40.38 | 116.39 | H1 |
| PI437776 | VIR 1302 | G. max | III | China | | 40.38 | 116.39 | H1 |
| PI437788A | VIR 3018 | G. max | II | China | | 40.38 | 116.39 | H1 |
| PI437793 | VIR 3024 | G. max | II | China | | 40.38 | 116.39 | H1 |
| PI437814A | An'da | G. max | II | China | Northeast | 40.38 | 116.39 | H1 |
| PI437991B | VIR 1657 | G. max | 0 | China | Northeast | 40.38 | 116.39 | H1 |
| PI438019B | VIR 1883 | G. max | II | China | Northeast | 40.38 | 116.39 | H1 |
| PI438083 | VIR 2506 | G. max | II | China | Northeast | 40.38 | 116.39 | H1 |
| PI438112B | VIR 2623 | G. max | III | China | Northeast | 40.38 | 116.39 | H1 |
| PI438239B | VIR 4536 | G. max | I | China | Northeast | 40.38 | 116.39 | H1 |
| PI438323 | Grignon 53-F-3 | G. max | I | France | | 48.83 | 2.22 | H1 |
| PI438335 | SAO 196-C | G. max | III | Algeria | | 26.66 | 2.14 | H1 |
| PI438336 | Sao 208 | G. max | 0 | Algeria | | 27.68 | 1.79 | H1 |
| PI438500 | Virginia | G. max | III | United States | | 38.67 | -90.4 | H1 |
| PI445824A | Wolfsthaler | G. max | 0 | Germany | | 52.51 | 13.41 | H1 |
| PI458510 | Ji Ti No. 1 | G. max | III | China | Liaoning | 41.2 | 122.34 | H1 |
| PI464912 | Dan Dou 1 | G. max | IV | China | Liaoning | 40.14 | 124.39 | H1 |
| PI464923 | Tie Fen 16 | G. max | I | China | Liaoning | 42.46 | 124.04 | H1 |
| PI467347 | Zi-hua-cuo-zi | G. max | II | China | Jilin | 44.23 | 126 | H1 |
| PI468408B | (Qi Huang No. 1) | G. max | III | China | | 40.38 | 116.39 | H1 |
| PI468908 | | G. max | 0 | China | Jilin | 44.23 | 126 | H1 |
| PI475820 | | G. max | II | China | Xinjiang | 46.86 | 83.23 | H1 |
| PI476352B | (Colnon) | G. max | II | Kyrgyzstan | Kirghiz SSR | 41.21 | 74.78 | H1 |
| PI479735 | Silihuang | G. max | III | China | Jilin | 43.63 | 126.5 | H1 |
| PI490766 | Dawudou | G. max | III | China | Hebei | 39.94 | 119.59 | H1 |
| PI495020 | Xu dou 2 | G. max | IV | China | Beijing | 40.38 | 116.39 | H1 |
| PI506933 | Kouiku 1 | G. max | IV | Japan | Kyushu and Okinav | 26.54 | 128.02 | H1 |
| PI506942 | Koushurei 235 | G. max | II | Japan | Tohoku | 39 | 141 | H1 |
| PI507293B | (Shoukin ou) | G. max | III | Japan | Hokuriku | 37 | 137.5 | H1 |
| PI507458 | Tousan kei BL 521 | G. max | IV | Japan | Kanto and Tosan | 36 | 139 | H1 |
| PI507467 | Tousan kei F 764 | G. max | IV | Japan | Kanto and Tosan | 36 | 139 | H1 |
| PI507471 | Tousan kei na 16 | G. max | III | Japan | Kanto and Tosan | 36 | 139 | H1 |

| | | | | | | | | | |
|---|---|---|---|---|---|---|---|---|---|
| PI507480 | Tousan kei YL 24 | G. max | IV | Japan | Kanto and Tosan | 36 | 139 | H1 | |
| PI518668 | TN 4-86 | G. max | IV | United States | Tennessee | 36.18 | -86.81 | H1 | |
| PI518727 | Ju huang | G. max | VI | China | Guangdong | 23.12 | 113.25 | H1 | |
| PI532463B | (He bei No. 1) | G. max | III | China | Hebei | 39.94 | 119.59 | H1 | |
| PI538386A | 1886 | G. max | III | China | Hebei | 39.94 | 119.59 | H1 | |
| PI548193 | T201 | G. max | IV | United States | Iowa | 41.56 | -93.5 | H1 | |
| PI548336 | Habaro | G. max | I | Russian Federatior | Khabarovsk | 48.5 | 135.13 | H1 | |
| PI548383 | Mansoy | G. max | III | China | Heilongjiang | 46.86 | 126.85 | H1 | |
| PI548411 | Seneca | G. max | II | China | Northeast China | 40.38 | 116.39 | H1 | |
| PI548447 | Cherokee | G. max | VIII | China | Zhejiang | 30.27 | 120.08 | H1 | |
| PI548452 | Dixie | G. max | V | Korea, North | Pyongyang | 39.04 | 125.75 | H1 | |
| PI548473 | Monetta | G. max | VII | China | Jiangsu | 32.06 | 118.85 | H1 | |
| PI548474 | Nanda | G. max | VIII | Korea, North | Hwanghae Puk | 38.51 | 125.76 | H1 | |
| PI548479 | Ototan | G. max | VIII | Taiwan | Taiwan | 24.73 | 121.08 | H1 | |
| PI548490 | Tanner | G. max | VII | Taiwan | Taiwan | 24.73 | 121.08 | H1 | |
| PI548521 | BSR 201 | G. max | II | United States | Iowa | 41.56 | -93.5 | H1 | |
| PI548561 | Hodgson | G. max | I | United States | Minnesota | 45.18 | -93.36 | H1 | |
| PI548571 | Harlon | G. max | I | Canada | Ontario | 43.69 | -79.4 | H1 | |
| PI548633 | Wye | G. max | IV | United States | Maryland | 39.29 | -76.7 | H1 | |
| PI548978 | Gail | G. max | VI | United States | Texas | 29.73 | -95.58 | H1 | |
| PI549017 | ZYD 3938 | G. max | IV | China | Ningxia | 37.33 | 106.04 | H1 | |
| PI549028 | Feng da li | G. max | V | China | Liaoning | 41.2 | 122.34 | H1 | |
| PI561371 | Fen dou 15 | G. max | IV | China | Shanxi | 37.33 | 111.83 | H1 | |
| PI561387 | Kosuzu | G. max | V | Japan | Tokyo | 35.73 | 139.57 | H1 | |
| PI567225 | Kisinevskaja 90 | G. max | 0 | Moldova | | 47 | 29 | H1 | |
| PI567226 | Har'kovskaja Zerno | G. max | 0 | Russian Federation | | 52.98 | 127.36 | H1 | |
| PI567231 | WJK-PRC-46 | G. max | VIII | China | Sichuan | 30.59 | 104.1 | H1 | |
| PI567307 | Hei huang dou | G. max | IV | China | Gansu | 36.03 | 103.84 | H1 | |
| PI567346 | Niu mao huang dou | G. max | V | China | Gansu | 36.03 | 103.84 | H1 | |
| PI567352A | Yang yan qing dou | G. max | IV | China | Gansu | 36.03 | 103.84 | H1 | |
| PI567353 | Yang yan ren dou | G. max | IV | China | Gansu | 36.03 | 103.84 | H1 | |
| PI567410B | (Yang huang dou) | G. max | VII | China | Shaanxi | 37.53 | 107.4 | H1 | |
| PI567415A | Bai da huang dou | G. max | IV | China | Shanxi | 37.33 | 111.83 | H1 | |
| PI567416 | Bai dou | G. max | IV | China | Shanxi | 37.33 | 111.83 | H1 | |
| PI567418A | Bai hei dou | G. max | II | China | Shanxi | 37.33 | 111.83 | H1 | |
| PI567426 | Bai huang dou | G. max | IV | China | Shanxi | 37.33 | 111.83 | H1 | |
| PI567428 | Bai ji yao | G. max | IV | China | Shanxi | 37.33 | 111.83 | H1 | |
| PI567435B | (Hei hei dou) | G. max | III | China | Shanxi | 37.33 | 111.83 | H1 | |
| PI567439 | Hong jia huang dou | G. max | V | China | Shanxi | 37.33 | 111.83 | H1 | |
| PI567489A | Er da li huang dou | G. max | IV | China | Hebei | 39.94 | 119.59 | H1 | |
| PI567532 | Dai ye xiao huang d | G. max | IV | China | Shandong | 36.6 | 118.97 | H1 | |
| PI567576 | Ping ding huang | G. max | III | China | Shandong | 36.6 | 118.97 | H1 | |
| PI567604A | Xin huang dou | G. max | IV | China | Shandong | 36.6 | 118.97 | H1 | |
| PI567675 | Yu cheng xiao tie jia | G. max | IV | China | Henan | 34.74 | 113.66 | H1 | |
| PI567698A | Fu yang (17) | G. max | IV | China | Anhui | 31.82 | 117.15 | H1 | |
| PI567746 | Pei xian da bai jiao | G. max | IV | China | Jiangsu | 32.06 | 118.85 | H1 | |
| PI567788 | Bienville | G. max | VIII | United States | Louisiana | 30.4 | -91.24 | H1 | |

| | | | | | | | | |
|---|---|---|---|---|---|---|---|---|
| PI574477 | Fen dou 31 | G.  max | IV | China | Shanxi | 37.33 | 111.83 | H1 |
| PI578375B | (Aan tu dang di hei | G.  max | I | China | | 40.38 | 116.39 | H1 |
| PI578412 | Gong jiao 6308-1 | G.  max | II | China | | 40.38 | 116.39 | H1 |
| PI578495 | Jin dou No. 4 | G.  max | IV | China | Beijing | 40.38 | 116.39 | H1 |
| PI578503 | Tie jia si li huang | G.  max | I | China | | 40.38 | 116.39 | H1 |
| PI587588B | (Tai xing niu mao h | G.  max | V | China | Jiangsu | 32.06 | 118.85 | H1 |
| PI587712B | (E dou No. 1) | G.  max | V | China | Hubei | 30.59 | 114.16 | H1 |
| PI587804 | Jing 789 | G.  max | IV | China | Hubei | 30.59 | 114.16 | H1 |
| PI587811A | ZDD005777 | G.  max | VIII | China | Hubei | 30.59 | 114.16 | H1 |
| PI592523 | Glacier | G.  max | 0 | United States | Minnesota | 45.18 | -93.36 | H1 |
| PI592937 | Jin dou 14 | G.  max | IV | China | | 40.38 | 116.39 | H1 |
| PI592940 | Jin dou 17 | G.  max | IV | China | | 40.38 | 116.39 | H1 |
| PI592952 | Zheng 77249 | G.  max | III | China | | 40.38 | 116.39 | H1 |
| PI592954 | Nin zhen No. 1 | G.  max | II | China | | 40.38 | 116.39 | H1 |
| PI592960 | Dong nong 38 | G.  max | I | China | Heilongjiang | 46.86 | 126.85 | H1 |
| PI593953 | Sui nong No. 10 | G.  max | I | China | | 40.38 | 116.39 | H1 |
| PI594170B | (Geden shirazu) | G.  max | I | Japan | Akita | 39.57 | 140.42 | H1 |
| PI594456A | Xiao jin huang | G.  max | III | China | Sichuan | 30.59 | 104.1 | H1 |
| PI594880 | Song zi dou | G.  max | V | China | Yunnan | 24.9 | 102.96 | H1 |
| PI594922 | Graham | G.  max | V | United States | North Carolina | 35.22 | -80.76 | H1 |
| PI597476 | Deogyukong | G.  max | V | Korea, South | Korea | 35.87 | 128.68 | H1 |
| PI597478B | (Paldalkong) | G.  max | III | Korea, South | Korea | 35.87 | 128.68 | H1 |
| PI598358 | TN 5-95 | G.  max | V | United States | Tennessee | 36.18 | -86.81 | H1 |
| PI602993 | Pi xian ruan tiao zh | G.  max | IV | China | Jiangsu | 32.06 | 118.85 | H1 |
| PI603290 | Zao shu 18 | G.  max | I | China | | 40.38 | 116.39 | H1 |
| PI603345 | ZDD00403 | G.  max | II | China | | 40.38 | 116.39 | H1 |
| PI603389 | Huang ke | G.  max | II | China | | 40.38 | 116.39 | H1 |
| PI603397 | Hei qi huang da dou | G.  max | IV | China | | 40.38 | 116.39 | H1 |
| PI603442 | Ke qi xiao hei dou | G.  max | III | China | | 40.38 | 116.39 | H1 |
| PI603458A | Shui dou | G.  max | IV | China | | 40.38 | 116.39 | H1 |
| PI603463 | Dong jie No. 1 | G.  max | II | China | | 40.38 | 116.39 | H1 |
| PI603488 | ZDD19294 | G.  max | III | China | | 40.38 | 116.39 | H1 |
| PI603492 | Qi hei dou | G.  max | IV | China | | 40.38 | 116.39 | H1 |
| PI603494 | Hai dou zi | G.  max | IV | China | | 40.38 | 116.39 | H1 |
| PI603495B | (Hong mi lan dou zi | G.  max | V | China | | 40.38 | 116.39 | H1 |
| PI603549 | Mei dou | G.  max | III | China | | 40.38 | 116.39 | H1 |
| PI603555 | Hua da hei dou | G.  max | IV | China | | 40.38 | 116.39 | H1 |
| PI603556 | ZDD08563 | G.  max | III | China | | 40.38 | 116.39 | H1 |
| PI603559 | ZDD08590 | G.  max | IV | China | | 40.38 | 116.39 | H1 |
| PI612730 | Zhong huong No. 1 | G.  max | II | China | | 40.38 | 116.39 | H1 |
| PI612754 | ZY 645 | G.  max | I | China | | 40.38 | 116.39 | H1 |
| PI59845 | Sohgetsu | G.  max | VI | Japan | Akita | 39.45 | 140.48 | H1 |
| PI458828 | Qun Xuan No.1 | G.  max | II | China | Jilin | 44.23 | 126 | H1 |
| PI556949 | Ke Feng No.1 | G.  max | IV | China | Beijing | 40.38 | 116.39 | H1 |
| PI547403 | L60-246 | G.  max | IV | United States | Illinois | 39.76 | -89.57 | H1 |
| PI490768 | Rao Shan Gun | G.  max | III | China | Hebei | 39.94 | 119.59 | H1 |
| PI603294 | Jin Yuan No.2 | G.  max | 0 | China | Heilongjiang | 46.86 | 126.85 | H1 |

| | | | | | | | | |
|---|---|---|---|---|---|---|---|---|
| PI578360 | Guan Nan Chun He | G. max | II | China | Jiangsu | 32.06 | 118.85 | H1 |
| PI283327 | Pingtung Pearl | G. max | V | Taiwan | Taiwan | 24.73 | 121.08 | H1 |
| PI578494A | Jin Dou No.1 | G. max | IV | China | Shanxi | 37.33 | 111.83 | H1 |
| PI297543 | Peking | G. max | II | China | North | 46.86 | 126.85 | H1 |
| PI567756 | Pei Xian Si Li Cao | G. max | IV | China | Jiangsu | 32.06 | 118.85 | H1 |
| PI603469 | Pa Man Qing | G. max | IV | China | Shandong | 36.6 | 118.97 | H1 |
| PI567164 | He Long You Tai | G. max | 0 | China | Jilin | 44.23 | 126 | H1 |
| PI317334B | (Kitamishiro) | G. max | II | Japan | Hokkaido | 43.46 | 142.61 | H1 |
| PI423766 | KAS 230-4 | G. max | IV | Korea, South | Kangwon | 37.17 | 128.5 | H1 |
| PI547612 | L74-826 | G. max | IV | United States | Illinois | 39.76 | -89.57 | H1 |
| PI567414 | Zhu Ye Qing | G. max | V | China | Shaanxi | 37.53 | 107.4 | H1 |
| | | | | | | | | |
| PI507088 | Nattou Kotsubu | G. max | VI | Japan | Kanto and Tosan | 36 | 139 | H2 |
| PI602502B | (Xiong yue xiao hua | G. max | IV | China | | 40.38 | 116.39 | H2 |
| | | | | | | | | |
| PI196175 | Yu tae | G. max | V | Korea, South | Korea | 35.87 | 128.68 | H3 |
| PI323576 | H 67-27 | G. max | IX | India | Uttar Pradesh | 29.77 | 79.77 | H3 |
| PI398610 | KAS 390-8 | G. max | V | Korea, South | Chungchong Puk | 37 | 128.17 | H3 |
| PI407788A | ORD 8113 | G. max | IV | Korea, South | Kyonggi | 37.83 | 127.51 | H3 |
| PI407965 | KAERI 504-4 | G. max | V | Korea, South | Cholla Nam | 35.23 | 126.31 | H3 |
| PI603175 | GL 2688 /96 | G. max | IV | Korea, North | | 39.04 | 125.75 | H3 |
| PI81785 | Chusei Hadaka | G. max | III | Japan | Hokkaido | 43.07 | 141.3 | H3 |
| PI166105 | Bhart | G. max | VII | India | Uttar Pradesh | 30.25 | 79.33 | H3 |
| PI391577 | Ch'a ye sheng tou | G. max | II | China | Jilin | 44.23 | 126 | H3 |
| PI437165A | Toncostebel'naja 2 | G. max | I | Russian Federation | Krasnodar | 45.1 | 39.27 | H3 |
| PI497953 | I.C. 192 | G. max | IX | India | Bihar | 25.98 | 85.68 | H3 |
| PI603722 | Nan chong ba yue h | G. max | VIII | China | | 40.38 | 116.39 | H3 |
| PI398296 | KAS 173-3 | G. max | II | Korea, South | Kyonggi | 37.14 | 127.07 | H3 |
| PI404166 | Krasnoarmejskaja | G. max | III | China | | 40.38 | 116.39 | H3 |
| PI404182 | Sin i tu li rau | G. max | III | China | China | 40.38 | 116.39 | H3 |
| PI404198B | (Sun huan do) | G. max | IV | China | | 40.38 | 116.39 | H3 |
| PI407729 | | G. max | IV | China | Beijing | 40.38 | 116.39 | H3 |
| PI417091 | Kuro mame | G. max | II | Japan | Kanto and Tosan re | 36 | 139 | H3 |
| PI424298 | KAS 300-10 | G. max | IV | Korea, South | Chungchong Nam | 36.33 | 127.42 | H3 |
| PI424391 | KAS 521-15 | G. max | VI | Korea, South | Cholla Puk | 35.63 | 127.25 | H3 |
| PI437654 | Er-hej-jan | G. max | III | China | China | 40.38 | 116.39 | H3 |
| PI467312 | Cha-mo-shi-dou | G. max | II | China | Jilin | 44.23 | 126 | H3 |
| PI548415 | Sooty | G. max | IV | China | Zhejiang | 30.27 | 120.08 | H3 |
| PI549031 | | G. max | III | China | Beijing | 40.19 | 116.2 | H3 |
| PI567171 | Hei he No. 1 | G. max | 0 | China | Heilongjiang | 46.86 | 126.85 | H3 |
| PI567258 | He pi dou | G. max | II | China | Jiangxi | 28.68 | 115.96 | H3 |
| PI567305 | Hei dou zi | G. max | IV | China | Gansu | 36.03 | 103.84 | H3 |
| PI567336B | (Lao hei dou) | G. max | IV | China | Gansu | 36.03 | 103.84 | H3 |
| PI567343 | Ma huang dou | G. max | V | China | Gansu | 36.03 | 103.84 | H3 |
| PI567516C | (Ba yue zha) | G. max | IV | China | Shandong | 36.6 | 118.97 | H3 |
| PI594012 | Heuksatangdu | G. max | V | Korea, South | Korea | 35.87 | 128.68 | H3 |
| PI594451 | Liu yue bao | G. max | III | China | Sichuan | 30.59 | 104.1 | H3 |

| | | | | | | | | |
|---|---|---|---|---|---|---|---|---|
| PI603176A | | G. max | IV | Korea, North | | 39.04 | 125.75 | H3 |
| PI603420 | ZDD01501 | G. max | II | China | Innermongolia | 40.8 | 111.87 | H3 |
| PI603424A | ZDD007871 | G. max | 0 | China | China | 40.38 | 116.39 | H3 |
| PI603497 | Hua dou | G. max | III | China | | 40.38 | 116.39 | H3 |
| PI605869A | Sample 140 | G. max | V | Vietnam | Lao Cai | 21 | 103.5 | H3 |
| PI612611 | Browngilgun | G. max | III | Korea, North | | 39.04 | 125.75 | H3 |
| PI86006 | Kiio Shokuzu | G. max | III | Japan | Hokkaido | 42.92 | 143.2 | H3 |
| PI87631-1 | Kindaizu | G. max | III | Japan | Saitama | 36.13 | 139.38 | H3 |
| PI89772 | 7193 | G. max | IV | China | | 40.38 | 116.39 | H3 |
| PI68604-1 | 285 | G. max | Unknov | China | Yaomyn, Manchuri | 46.2 | 126.07 | H3 |
| PI89005-5 | 5950 | G. max | Unknov | China | Manchuria | 46.2 | 126.07 | H3 |
| PI89775 | 7221 | G. max | VI | China | Near Fa Hua Ssu te | 24.65 | 113.64 | H3 |
| PI103088 | Ming Chuan | G. max | III | China | Henan | 34.74 | 113.66 | H3 |
| PI171428 | Large Yellow Soybe | G. max | IV | China | Beijing | 39.76 | 116.54 | H3 |
| PI171451 | Kosamame | G. max | VII | Japan | Kanagawa | 35.59 | 139.35 | H3 |
| PI342619A | | G. max | 0 | Russian Federatior | Primorye | 45 | 135 | H3 |
| PI361093 | Novosadska Br. 1 | G. max | I | Serbia | | 44.02 | 21.15 | H3 |
| PI398633 | KAS 390-17-2 | G. max | V | Korea, South | Chungchong Puk | 37 | 128.17 | H3 |
| PI407708A | Feng shou No. 10 | G. max | 0 | China | Heilongjiang | 46.86 | 126.85 | H3 |
| PI417381 | Tenpoku shirome | G. max | 0 | Japan | Hokkaido | 43.46 | 142.61 | H3 |
| PI417581 | H-060072 | G. max | V | United States | | 38.67 | -90.4 | H3 |
| PI437110A | VIR 244 | G. max | III | Russian Federatior | Jewish Region, Far | 48.6 | 132.3 | H3 |
| PI437838 | DV-254 | G. max | II | Russian Federation | | 52.98 | 127.36 | H3 |
| PI438230A | VIR 4521 | G. max | I | China | Northeast | 40.38 | 116.39 | H3 |
| PI438309 | VIR 3017 | G. max | I | China | | 40.38 | 116.39 | H3 |
| PI438496C | (Peking) | G. max | IV | United States | | 38.67 | -90.4 | H3 |
| PI464896 | Jou Nong No. 5 | G. max | I | China | Jilin | 44.23 | 126 | H3 |
| PI497964A | I.C. 9461 | G. max | Unknov | India | Sikkim | 27.33 | 88.54 | H3 |
| PI497967 | PLSO 96 | G. max | VII | India | Jammu and Kashm | 33.89 | 76.66 | H3 |
| PI514671 | Feng shou No. 7 | G. max | 0 | China | Heilongjiang | 46.86 | 126.85 | H3 |
| PI561318A | Hui nan bai hua xia | G. max | I | China | Beijing | 40.38 | 116.39 | H3 |
| PI567262A | Similar to: Gu tian t | G. max | II | China | Fujian | 26.06 | 119.39 | H3 |
| PI567407 | Xiao dou | G. max | V | China | Shaanxi | 37.53 | 107.4 | H3 |
| PI567408 | Xiao jin huang | G. max | V | China | Shaanxi | 37.53 | 107.4 | H3 |
| PI567488A | Di liu huang dou No | G. max | IV | China | Hebei | 39.94 | 119.59 | H3 |
| PI567548 | Hua li hu zi | G. max | IV | China | Shandong | 36.6 | 118.97 | H3 |
| PI567780B | (Tong shan zheng ji | G. max | IV | China | Jiangsu | 32.06 | 118.85 | H3 |
| PI603162 | GL 2631 /96 | G. max | IV | Korea, North | | 39.04 | 125.75 | H3 |
| PI603426G | (Ben di yuan huang | G. max | II | China | | 40.38 | 116.39 | H3 |
| PI603526 | Hei you dou | G. max | IV | China | | 40.38 | 116.39 | H3 |
| PI385942 | Enrei | G. max | IV | Japan | Nagano | 36.7 | 137.86 | H3 |
| | | | | | | | | |
| PI594629 | Xiao hua lian | G. max | VI | China | Guizhou | 26.65 | 106.69 | H4 |
| PI79691-4 | | G. max | III | China | Heilongjiang | 45.75 | 126.65 | H4 |
| PI361066B | (F. 56-17) | G. max | I | Romania | | 44.42 | 26.11 | H4 |
| | | | | | | | | |
| PI366123 | | G. soja | IV | China | Ningxia | 37.33 | 106.04 | H5 |

| | | | | | | | | |
|---|---|---|---|---|---|---|---|---|
| PI378692 | | G. soja | V | China | Shaanxi | 34.88 | 110.01 | H5 |
| PI407038 | RB 1072 | G. soja | VII | Japan | Fukuoka | 33.2 | 130.37 | H5 |
| | | | | | | | | |
| PI407096 | RB 1072 | G. soja | V | Japan | Akita | 39.7 | 140.73 | H6 |
| PI407162 | K1-D | G. soja | V | China | Jiangsu | 32.06 | 118.85 | H6 |
| PI407300 | | G. soja | 0 | China | Heilongjiang | 48.48 | 127.97 | H6 |
| PI424025B | 74023 | G. soja | 0 | Russian Federatior | Primorye | 45 | 135 | H6 |
| PI424088 | 74088 | G. soja | VI | China | Jiangsu | 32.06 | 118.85 | H6 |
| PI468400A | | G. soja | 0 | Russian Federatior | Amur | 64.41 | 144.03 | H6 |
| PI479769 | Long 79-5801 | G. soja | 0 | Russian Federatior | Amur | 52.98 | 127.36 | H6 |
| PI483465 | | G. soja | IV | Japan | Fukushima | 37.46 | 139.84 | H6 |
| PI507618 | NIAR 040015 | G. soja | VII | Japan | Hyogo | 34.92 | 135.23 | H6 |
| PI507641 | NIAR 060014 | G. soja | IV | Korea, South | Kyonggi | 37.28 | 127.11 | H6 |
| PI522226 | VIR 8511 | G. soja | V | Korea, South | Kyonggi | 37.9 | 127.2 | H6 |
| | | | | | | | | |
| PI407157 | RB 1072 | G. soja | IV | Japan | Iwate | 39.7 | 141.25 | H7 |
| PI407243 | K36-B | G. soja | IV | Japan | Iwate | 39.7 | 141.2 | H7 |
| PI407303 | | G. soja | V | Japan | Iwate | 39.72 | 141.14 | H7 |
| PI507630 | NIAR 060002 | G. soja | V | Japan | Hyogo | 35.4 | 134.77 | H7 |
| PI507757 | VIR 8451 | G. soja | VI | Japan | Saitama | 36.03 | 139.53 | H7 |
| PI378699A | | G. soja | VI | Japan | Chiba | 35.6 | 140.12 | H7 |
| PI507609 | NIAR 040023 | G. soja | VII | Japan | Nara | 34.53 | 135.95 | H7 |
| PI507615 | NIAR 040012 | G. soja | VII | Japan | Tokyo | 35.73 | 139.57 | H7 |
| PI507638 | NIAR 060010 | G. soja | VI | Japan | Tochigi | 36.55 | 139.73 | H7 |
| PI507830B | VIR 9044 | G. soja | VI | Japan | Hyogo | 34.79 | 134.85 | H7 |
| PI378690 | | G. soja | V | Japan | Nagano | 36.7 | 137.86 | H7 |
| | | | | | | | | |
| PI366120 | | G. soja | IV | Japan | Akita | 39.53 | 140.38 | H8 |
| PI378697A | | G. soja | V | Japan | Aomori | 40.6 | 140.47 | H8 |
| | | | | | | | | |
| PI407042 | RB 1072 | G. soja | V | Korea, South | Kyonggi | 37.21 | 126.82 | H9 |
| PI593983 | Hidaka-6 | G. soja | V | Korea, South | Kyonggi | 37.9 | 126.98 | H9 |
| PI407220 | K25-B | G. soja | V | Korea, South | Kyongsang Puk | 36.5 | 128.15 | H9 |
| PI562551 | KC26 | G. soja | Unknow | Korea, South | Cholla Puk | 35.82 | 127.12 | H9 |
| | | | | | | | | |
| PI407202 | K15 | G. soja | V | Korea, South | Chungchong Nam | 36.57 | 126.68 | H10 |
| PI507619B | NIAR 040016 | G. soja | VI | Japan | Shimane | 35.12 | 132.49 | H10 |
| PI562534 | KA5 | G. soja | V | Korea, South | Kangwon | 37.5 | 127.98 | H10 |
| PI562557 | KE10 | G. soja | VI | Japan | Nagano | 36.7 | 137.86 | H10 |
| PI562559 | KE16 | G. soja | Unknow | Korea, South | Kyonggi | 37.23 | 126.93 | H10 |
| PI562565 | KF13 | G. soja | Unknow | Korea, South | Cholla Puk | 35.82 | 127.12 | H10 |
| | | | | | | | | |
| PI549032 | ZYD 2632 | G. soja | V | Korea, South | Kangwon | 37.26 | 128.42 | H11 |
| PI407083 | RB 1072 | G. soja | V | Korea, South | Kyongsang Puk | 35.6 | 128.75 | H11 |
| | | | | | | | | |
| PI464890B | (Gong di No. 2019) | G. soja | V | Korea, South | Kyongsang Puk | 35.68 | 128.75 | H12 |
| PI424107A | 74106 | G. soja | VII | Japan | Nagasaki | 33 | 129.5 | H12 |

| | | | | | | | | |
|---|---|---|---|---|---|---|---|---|
| | | | | | | | | |
| PI532450 | GD50344 | G. soja | III | Japan | Hokkaido | 42.87 | 142.44 | H13 |
| PI407288 | | G. soja | V | Korea, South | Chungchong Puk | 36.95 | 127.74 | H13 |
| | | | | | | | | |
| PI407315 | | G. soja | III | China | Liaoning | 40.55 | 124.07 | H14 |
| PI407077 | RB 1072 | G. soja | II | China | Jilin | 43.51 | 124.81 | H14 |
| | | | | | | | | |
| PI407248 | K37-D | G. soja | VI | Japan | Aichi | 34.94 | 137.24 | H15 |
| PI507656 | NIAR 090011 | G. soja | VII | Japan | Aichi | 34.94 | 137.24 | H15 |
| | | | | | | | | |
| PI468916 | | G. soja | III | China | Liaoning | 41.2 | 122.34 | H16 |
| PI468918 | | G. soja | III | China | Liaoning | 41.2 | 122.34 | H16 |
| | | | | | | | | |
| PI101404A | | G. soja | III | Russian Federatior | Primorye | 45 | 135 | H17 |
| PI424097 | 74096 | G. soja | II | China | Heilongjiang | 46.2 | 126.07 | H17 |
| | | | | | | | | |
| PI479751 | GD 50351-1 | G. soja | III | China | Jilin | 44.23 | 126 | H18 |
| PI479752 | GD 50388-2 | G. soja | 0 | China | Heilongjiang | 46.86 | 126.85 | H18 |
| PI479768 | Long 79-3313-1 | G. soja | I | Russian Federatior | Amur | 52.98 | 127.36 | H18 |
| PI507761 | VIR 8455 | G. soja | I | Russian Federatior | Primorye | 45 | 135 | H18 |
| PI522228 | VIR 8514 | G. soja | Unknow | Russian Federatior | Khabarovsk | 48.5 | 135.13 | H18 |
| PI578341 | L 521 | G. soja | I | Russian Federatior | Primorye | 45 | 135 | H18 |
| PI342622A | | G. soja | V | Korea, South | Kyonggi | 37.21 | 126.99 | H18 |
| PI407190 | K56-C | G. soja | II | China | Jilin | 44.23 | 126 | H18 |
| | | | | | | | | |
| PI479746B | GD 50062 | G. soja | I | China | Jilin | 44.23 | 126 | H19 |
| PI407275 | K101-A | G. soja | 0 | China | Nei Monggol | 42.82 | 113.29 | H19 |
