# Supplementary material for: Selection of GmSWEET39 for oil and protein improvement in soybean
Source: PLoS Genet. 2020 Nov 11;16(11):e1009114. doi: 10.1371/journal.pgen.1009114 (PMC7721174; doi:10.1371/journal.pgen.1009114)
Supplement: S4 Table — (PDF) [file pgen.1009114.s006.pdf]

S4 Table Information of the 75 landrace and milestone lines used in this study

Name	PI ID	Subspecies	Variety	Year Introduced or Released	Maturity Group	CC-deletion
Haberlandt	PI548456	G. max	Landrace	1907	VI	yes
Tokyo	PI548493	G. max	Landrace	1907	VII	yes
Manchu	PI548365	G. max	Landrace	1918	III	yes
Mandarin	PI548378	G. max	Landrace	1920	I	yes
Dunfield	PI548318	G. max	Landrace	1923	III	yes
Illini	PI548348	G. max	Landrace	1927	III	yes
No. 94	PI71506	G. max	Landrace	1927	IV	yes
5913	PI88788	G. max	Landrace	1930	III	yes
Mukden	PI548391	G. max	Landrace	1932	II	yes
Mandarin (Ottawa)	PI548379	G. max	Landrace	1934	0	yes
Arksoy	PI548438	G. max	Landrace	1937	VI	yes
Richland	PI548406	G. max	Landrace	1938	II	yes
A.K. (Harrow)	PI548298	G. max	Landrace	1939	III	yes
Ralsoy	PI548484	G. max	Landrace	1940	VI	yes
CNS	PI548445	G. max	Landrace	1943	VII	yes
S-100	PI548488	G. max	Landrace	1945	V	yes
Roanoke	PI548485	G. max	Landrace	1946	VII	yes
FC 31745	FC31745	G. max	Landrace	1948	VI	yes
No. 3226 Brown	PI171442	G. max	Landrace	1948	V	yes
Ogden	PI548477	G. max	Milestone	1940	VI	yes
Volstate	PI548494	G. max	Milestone	1942	VII	yes
Capital	PI548311	G. max	Milestone	1944	0	yes
Adams	PI548502	G. max	Milestone	1948	III	yes
Blackhawk	PI548516	G. max	Milestone	1950	I	yes
Harosoy	PI548573	G. max	Milestone	1951	II	yes
Perry	PI548603	G. max	Milestone	1951	IV	yes
Dorman	PI548653	G. max	Milestone	1952	V	yes
Clark	PI548533	G. max	Milestone	1953	IV	yes
Hill	PI548654	G. max	Milestone	1953	V	yes
Jackson	PI548657	G. max	Milestone	1953	VII	yes
Chippewa	PI548530	G. max	Milestone	1954	I	yes
Lee	PI548656	G. max	Milestone	1954	VI	yes
Ford	PI548562	G. max	Milestone	1958	III	yes
Hood	PI548980	G. max	Milestone	1958	VI	yes
Shelby	PI548574	G. max	Milestone	1958	III	yes
Merit	PI548545	G. max	Milestone	1959	0	yes
Kent	PI548586	G. max	Milestone	1961	IV	yes
Bragg	PI548660	G. max	Milestone	1963	VII	yes
Wayne	PI548628	G. max	Milestone	1964	III	yes
Amsoy	PI548506	G. max	Milestone	1965	II	yes
Dare	PI548987	G. max	Milestone	1965	V	yes
Davis	PI553039	G. max	Milestone	1965	VI	yes
Pickett	PI548988	G. max	Milestone	1965	VI	yes
Corsoy	PI548540	G. max	Milestone	1967	II	yes
Beeson	PI548510	G. max	Milestone	1968	II	yes
Calland	PI548527	G. max	Milestone	1968	III	yes
Ransom	PI548989	G. max	Milestone	1970	VII	yes
Bonus	PI548517	G. max	Milestone	1971	IV	yes
Williams	PI548631	G. max	Milestone	1971	III	yes
Essex	PI548667	G. max	Milestone	1972	V	yes
Tracy	PI548983	G. max	Milestone	1973	VI	yes
Woodworth	PI548632	G. max	Milestone	1974	III	yes
Harcor	PI548570	G. max	Milestone	1975	II	yes
Centennial	PI548975	G. max	Milestone	1976	VI	yes

GaSoy17	PI553046	G. max	Milestone	1977	VII	yes
Cumberland	PI548542	G. max	Milestone	1978	III	yes
Oakland	PI548543	G. max	Milestone	1978	III	yes
Amcor	PI548505	G. max	Milestone	1979	II	yes
Braxton	PI548659	G. max	Milestone	1979	VII	yes
Century	PI548512	G. max	Milestone	1979	II	yes
Pella	PI548523	G. max	Milestone	1979	III	yes
Douglas	PI548555	G. max	Milestone	1980	IV	yes
Lawrence	PI518673	G. max	Milestone	1981	IV	yes
Williams 82	PI518671	G. max	Milestone	1981	III	yes
Young	PI508266	G. max	Milestone	1984	VI	yes
Zane	PI548634	G. max	Milestone	1984	III	yes
Hutcheson	PI518664	G. max	Milestone	1987	V	yes
Jack	PI540556	G. max	N/A	1989	II	yes
Brim	PI548986	G. max	Milestone	1990	VI	yes
Hagood	PI555453	G. max	Milestone	1990	VII	yes
Cook	PI553045	G. max	Milestone	1991	VIII	yes
Dillon	PI592756	G. max	Milestone	1994	VI	yes
5601T	PI630984	G. max	Milestone	2001	V	yes
NC-Roy	PI617045	G. max	Milestone	2001	VI	yes
NC-Raleigh	PI641156	G. max	Milestone	2002	VII	yes
